# A Pyrimidine-Based Tubulin Inhibitor Shows Potent Anti-Glioblastoma Activity In Vitro and In Vivo

**DOI:** 10.3390/ph18121891

**Published:** 2025-12-15

**Authors:** Satyanarayana Pochampally, Lawrence M. Pfeffer, Gustavo A. Miranda-Carboni, Macey Daniel, Jazz I. James, Allana Smith, Chuan He Yang, Hannah R. Kelso, Deanna N. Parke, Dong-Jin Hwang, Wei Li, Duane D. Miller

**Affiliations:** 1 Department of Pharmaceutical Sciences, College of Pharmacy, University of Tennessee Health Science Center, Memphis, TN 38163, USA; spochamp@uthsc.edu (S.P.); dhwang@uthsc.edu (D.-J.H.); wli@uthsc.edu (W.L.); 2 Department of Pathology and Laboratory Medicine, Center for Cancer Research, College of Medicine, University of Tennessee Health Science Center, Memphis, TN 38163, USA; jazijame@uthsc.edu (J.I.J.); asmit611@uthsc.edu (A.S.); cyang@uthsc.edu (C.H.Y.); hanrkels@uthsc.edu (H.R.K.); dparke2@uthsc.edu (D.N.P.); 3 Department of Medicine, Center for Cancer Research, College of Medicine, University of Tennessee Health Science Center, Memphis, TN 38163, USA; gmirand1@uthsc.edu (G.A.M.-C.); khd927@uthsc.edu (M.D.)

**Keywords:** pyrimidine dihydroquinoxalinone, microtubule-targeting agents (MTAs), glioblastoma (GBM), cancer

## Abstract

**Background**: Glioblastoma (GBM) is an aggressive and treatment-resistant brain tumor with few effective therapies. Tubulin polymers are crucial for maintaining cell–cell signaling, cell proliferation, and cell division. Therefore, tubulin has been targeted by medicinal chemists to develop novel therapeutics to treat cancer. In this regard, we developed novel small-molecule tubulin inhibitors as potential therapeutics to treat GBM. **Methods**: We synthesized a focused library of pyrimidine-containing dihydroquinoxalinone-based analogs and tested nine compounds for cytotoxicity in GBM cell lines using the Sulforhodamine B (SRB) cell viability assay. We identified compound **8c** as the most promising compound and evaluated the in vitro effects of **8c** on GBM cell growth using live cell imaging and assessed apoptosis using a cell death ELISA. We then tested its anticancer activity in vivo on GBM xenografts grown in immunocompromised mice. **Results**: Several compounds demonstrated nanomolar IC_50_ values in cell viability assays and outperformed temozolomide (TMZ), the current standard treatment for GBM patients. We identified compound **8c**, which is a pyrimidine analog with a secondary amine, as the lead candidate for GBM studies in vitro and in vivo. Compound **8c** reduced cell viability in a dose-dependent manner and induced complete growth arrest within 48 h at 3–10 nM concentrations in GBM cell lines. ELISA confirmed that compound **8c** triggered dose-dependent apoptosis, whereas TMZ failed to induce apoptosis at nM concentrations. In vivo, compound **8c** significantly inhibited GBM xenograft growth in immunocompromised mice by 66%. **Conclusions**: The potent tubulin inhibitor compound **8c** has strong anti-GBM activity in vitro and in vivo and merits further preclinical development.

## 1. Introduction

Glioblastoma (GBM) is the most common and aggressive type of primary brain tumor, with approximately 12,000 new cases diagnosed annually in the United States [1]. Despite advances in surgical resection, radiotherapy, and chemotherapy, GBM remains highly lethal due to its rapid progression and resistance to conventional treatments. The median survival time for patients with GBM remains poor, typically ranging from 12 to 18 months [2]. Temozolomide (TMZ) is an oral DNA-alkylating agent that can cross the blood–brain barrier (BBB) and is the standard chemotherapeutic used in GBM treatment [2]. By interfering with DNA replication in cancer cells, TMZ induces cell death and helps slow tumor growth. However, its clinical effectiveness is often limited by the development of resistance mechanisms within tumors. As a result, TMZ rarely extends patient survival beyond 15 months [2]. The emergence of TMZ resistance also poses a major challenge in GBM therapy, highlighting the urgent need to develop novel, potent small molecules capable of effectively targeting GBM cells. Developing such agents to overcome the limitations of current treatments, particularly TMZ resistance, is a critical focus in GBM research.

Microtubule-targeting agents (MTAs) have become a significant area of focus in the development of anticancer drugs. Microtubules are dynamic heteropolymers composed of α- and β-tubulin subunits [3,4,5]. These structures play a pivotal role in various cellular processes, including maintaining cell shape, enabling intracellular transport, and facilitating cell division [6]. MTAs disrupt polymer dynamics, triggering cell cycle arrest that leads to cell death. Thus, microtubules are a good target for anticancer therapy [7,8,9,10,11,12]. Three major binding sites have been identified in tubulins: the taxane [13], vinca alkaloid [14], and colchicine sites [15]. A wide variety of small-molecule tubulin-binding agents have been developed as therapeutics for a variety of other cancers. A broad range of small-molecule tubulin-binding agents, including pyrimidine-containing hybrids [16] and heterocyclic-fused pyrimidine scaffolds such as isoxazole, thiazole, 1,3-thiazine, and thiazolopyrimidine derivatives, have been developed as potential therapeutics for various cancers [17,18,19,20]. Hence, developing novel compounds that can bind to the colchicine binding site and induce cell death is crucial for improving outcomes in GBM therapy. In this context, we have previously shown by X-ray crystallographic studies that compound **8c** binds to tubulin at the colchicine binding site. Furthermore, the antiproliferative potency of 8c against melanoma, breast, pancreatic, and prostate cancer cell lines demonstrated excellent potencies when compared to colchicine and paclitaxel.

**Figure 1:**
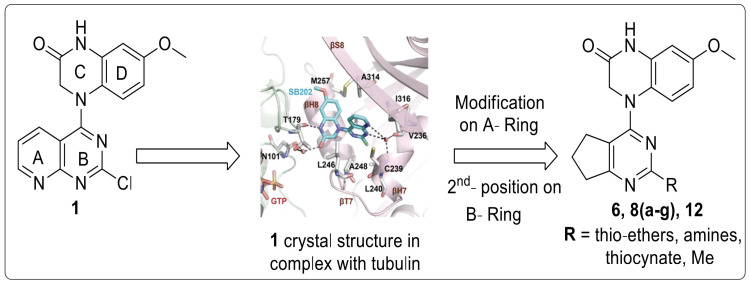
SAR of the selected lead molecule and its analogs.

Taken together this concludes that our molecules are evidently binding to the colchicine binding site of tubulin to exhibit antiproliferative properties [21,22,23,24]. In the current study, we sought to evaluate the anticancer activity of nine most potent microtubule-inhibiting molecules against glioblastoma cell lines for the first time. The crystal structure of heterocyclic-fused pyridopyrimidines (**1**), shown in Figure 1, revealed that this molecule binds strongly to β-Cys239 and β-Val236 through a water molecule-mediated H-bond and demonstrated significant potency against melanoma, prostate, lung, and pancreatic cancer in vitro [25]. However, compound **1** showed low to moderate metabolic stability, poor water solubility, and considerable in vivo toxicity. Therefore, we addressed these shortcomings through structural modifications.

## 2. Results

### 2.1. Chemistry

**Scheme 1:**
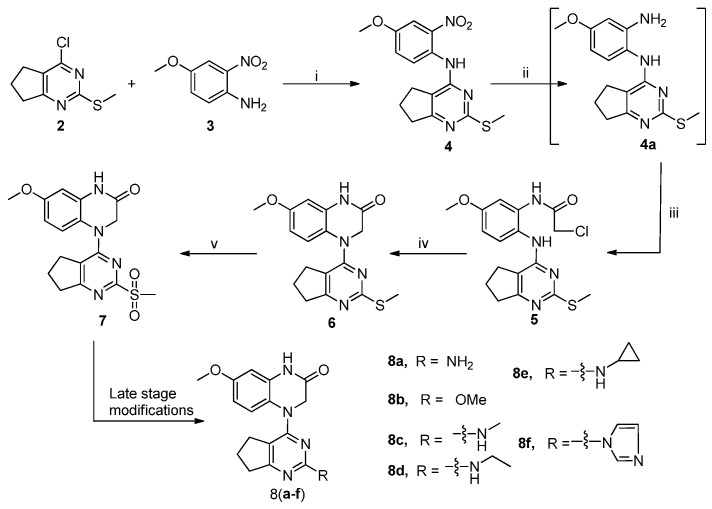
Design and synthesis of cyclopentyl-fused pyrimidine dihydroquinoxalinone (**8a–f**) analogs. Reagents and conditions: (i) IPA/HCl, 50 °C; (ii) Zn/AcOH, CH_2_Cl_2_; (iii) chloroacetyl chloride/K_2_CO_3_, acetone, 0 °C; (iv) 60% NaH, THF, 0 °C to room temperature; (v) oxone, methanol/water, room temperature.

**Scheme 2:**
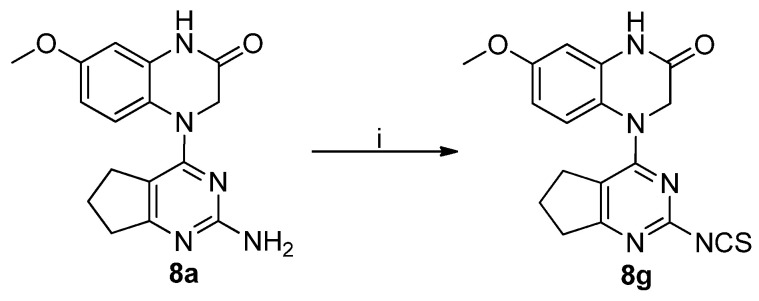
Design and synthesis of cyclopentyl-fused isothiocyanate pyrimidine dihydroquinoxalinone (**8g**) analog. Reagents and conditions: (i) thiophosgene, DIPA, CH_2_Cl_2_, 0 °C to room temperature.

**Scheme 3:**
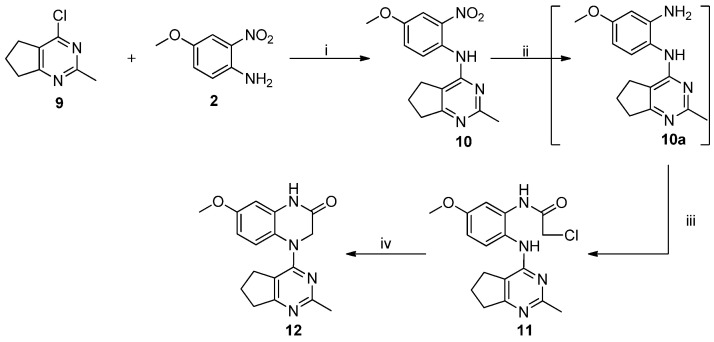
Design and synthesis of cyclopentyl-fused methyl pyrimidine dihydroquinoxalinone (**12**) analog. Reagents and conditions: (i) IPA/HCl, 50 °C; (ii) Zn/AcOH, CH_2_Cl_2_; (iii) chloroacetyl chloride/K_2_CO_3_, acetone, 0 °C to room temperature; (iv) 60% NaH, THF, 0 °C to room temperature.

Upon careful examination of the binding interactions of **1** with the colchicine binding site in the X-ray crystal structure, we identified that incorporating additional H-bond donor or acceptor in place of the Cl atom on the 2nd position on the B ring would potentially enhance binding and have a positive impact on toxicity and solubility, while retaining or even having improved potency. We incorporated various functional groups-such as amines, ethers, thioethers, and isothiocyanate at the 2nd position of the pyrimidine (B-ring), as illustrated in Scheme 1, Scheme 2 and Scheme 3 [21,22,23,24]. The key precursor compound **2** (PFI-3) was coupled with 4-methoxy-2-nitroaniline (compound **3**) in dry isopropyl alcohol (IPA) in the presence of a catalytic amount of HCl (3–4 drops) to afford 2-methylthio-4-(4-methoxy-2-nitrophenyl)aminopyrimidine (compound **4**). Subsequent reduction of compound **4** with zinc and catalytic acetic acid (AcOH) at 0 °C furnished the corresponding amine (compound **4a**), which was immediately reacted with chloroacetyl chloride to give compound **5**. Intramolecular cyclization of compound **5** in the presence of 60% sodium hydride (NaH) and anhydrous tetrahydrofuran (THF) yielded the six-membered dihydroquinoxalinone derivative (compound **6**). Alternatively, oxidation of intermediate **6** using oxone in a methanol/water (1:1) mixture produced the corresponding sulfone derivative (compound **7**). Finally, substitution of the sulfone group at the 2nd position of the pyrimidine ring with various nucleophiles—such as primary or secondary amines, heterocycles (e.g., imidazole), or alkoxides—afforded the final derivatives (**8a**–**f**, Scheme 1).

The amine and alkoxy derivatives **8**(**a,b**) were prepared using sodium azide (NaN_3_) and sodium methoxide (NaOMe) in methanol, respectively. Compounds **8**(**c**–**f**) were synthesized by reacting the key intermediate (**7**) with different amines, such as N-methylamine, N-ethylamine, N-cyclopropylamine, and imidazole. Finally, the isothiocyanate derivative (**8g**) was obtained by treating compound **8a** with thiophosgene in the presence of a base, as shown in Scheme 2 [21,23].

Based on the promising results of the amine and ether derivatives (compounds **6** and **8a**–**g**), we further explored alkyl substitution, specifically a methyl group at the 2nd position of the pyrimidine ring, to evaluate its effect on activity [24]. The methyl-substituted pyrimidine analogue (compound **12**) was synthesized from 4-chloro-2-methyl-6,7-dihydro-5H-cyclopenta[*d*]pyrimidine (compound **9**) and 4-methoxy-2-nitroaniline (compound **3**) in an acidic medium to give compound **10**. Reduction of compound **10** yielded the corresponding amine (compound **10a**), which was then reacted with chloroacetyl chloride to form compound **11**. Finally, intramolecular cyclization of compound **11** in the presence of 60% NaH and THF afforded the six-membered dihydroquinoxalinone compound **12**, as shown in Scheme 3 (Compound characterization data of the target compounds is present in Supplementary Materials).

### 2.2. MTAs Reduce GBM Cell Viability In Vitro

Over the past decade, our laboratory has developed a series of small-molecule tubulin inhibitors targeting the colchicine binding site, potentially offering therapeutic advantages over other MTAs [26,27,28,29]. To advance these compounds as pharmacological agents against the deadliest of gliomas, glioblastoma (GBM), we focused on our most potent derivatives containing pyrimidine and dihydroquinoxalinone moieties with diverse functional groups. From this library, nine analogs (compounds **6**, **8**(**a**–**g**), **12**, Table 1) were selected and tested for their effects on the viability of two GBM cell lines (MT330 and LN229) using the sulforhodamine B (SRB) assay, a standard method to measure cytotoxicity in cell-based assays. TMZ, the current chemotherapeutic drug used clinically, was included as an internal control.

**Figure 2:**
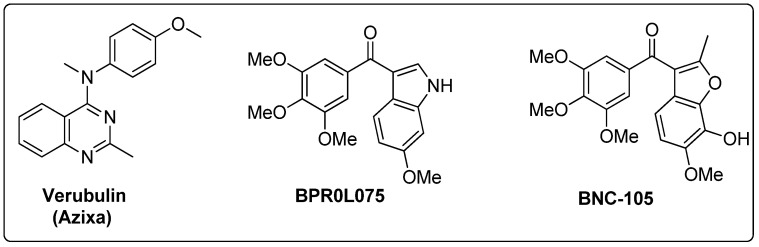
Previous tubulin-binding agents with preclinical efficacy in GBM.

Based on the structures of previously characterized compounds such as Verubulin, BPR0L075, and BNC105 (Figure 2), which demonstrated preclinical efficacy, we hypothesized that the methoxy (OMe) group on the aromatic ring is critical for biological potency. We selected compound **8b**, which contains this group, and found it exhibited moderate cytotoxic activity with IC_50_ values of 15.6 ± 1.38 and 80.1 ± 6.3 nM in the two glioma cell lines, respectively. Modification of the OMe group in compound **8b** to a thioether (SMe, compound **6**) markedly increased potency, with IC_50_ values of 1.88 ± 0.67 and 2.49 ± 0.98 nM in the respective cell lines.

Encouraged by these results, we then examined various pyrimidine analogs with primary (1°) and secondary (2°) amine substitutions at the 2nd position of the pyrimidine ring. Interestingly, secondary amine-bearing pyrimidine analogs showed strong potency. Compound **8c** exhibited IC_50_ values of 2.09 ± 0.59 and 2.36 ± 0.64 nM, compound **8d** had 3.1 ± 1.27 and 3.15 ± 1.48 nM, and compound **8e** showed 0.81 ± 0.28 and 5.82 ± 1.84 nM in the two glioma cell lines, respectively. In contrast, the primary amine analog (compound **8b**) was biologically inactive (IC_50_ > 1000 nM). Incorporation of nitrogen at the 2nd position as part of a heterocyclic ring improved potency, as demonstrated by compound **8**(**c**,**d**). Additionally, derivatives containing isothiocyanate and methyl groups (compounds **8g** and **12**) displayed significant cytotoxic activity (IC_50_ values ranging from 2.09 ± 0.43 to 5.71 ± 3.41 nM). The IC_50_ values for all compounds are summarized in Table 1.

**Figure 3:**
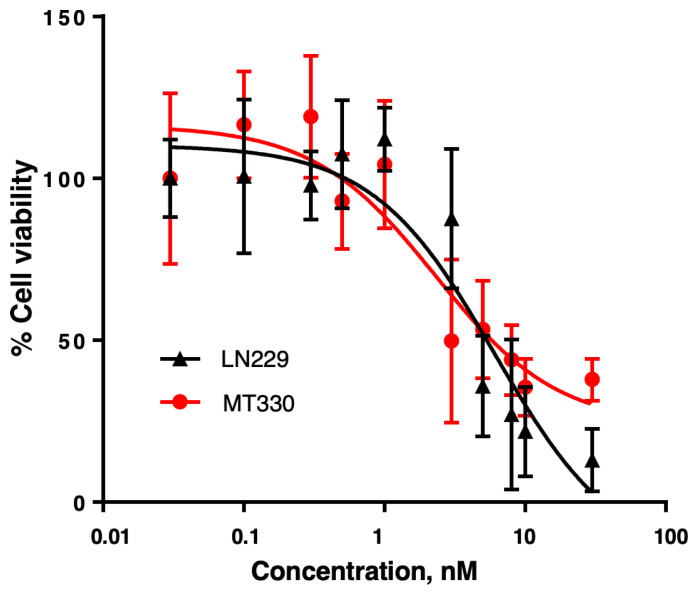
Compound **8c** reduces GBM cell viability in a dose-dependent manner. LN229 (black triangles) and U251 (red circles) GBM cells were treated with increasing concentrations of compound **8c** for 5 days, followed by assessment of cell viability using the sulforhodamine B (SRB) assay. Absorbance at 540 nm (A_540_) was measured and normalized to vehicle (DMSO)-treated controls. Both cell lines exhibited a dose-dependent decrease in viability, with MT330 cells showing slightly greater sensitivity to compound **8c**. Data are presented as mean ± SEM from three independent experiments.

For reference, TMZ, the standard DNA-alkylating agent used to treat GBM, required concentrations at least 1000-fold higher to induce cytotoxicity (IC_50_ of 20–400 µM). This study revealed that most of the tested compounds induced cytotoxicity in GBM cells with nanomolar potency. Based on these promising results, we selected compound **8c** for further investigation of its anticancer activity. The complete dose–response curve for compound **8c** in the SRB assay is shown in Figure 3.

### 2.3. Compound ***8c*** Reduces the Proliferation of GBM Cells in Vitro

**Figure 4:**
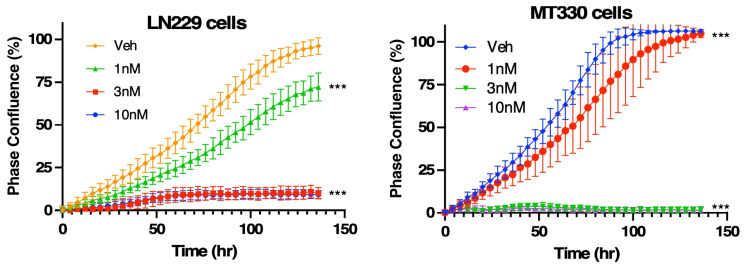
Compound **8c** inhibits the proliferation of GBM cells in a dose-dependent manner. LN229 (**left**) and MT330 (**right**) GBM cells were treated with vehicle (Veh) or compound **8c** at concentrations of 1, 3, or 10 nM. Cell proliferation was monitored in real time using the IncuCyte Live-Cell Imaging System, and phase confluence (%) was measured every 4 h over a 5-day period as an indicator of cell growth. Vehicle-treated cells entered exponential growth, while compound **8c** induced a dose-dependent reduction in proliferation in both cell lines. At 3 and 10 nM, compound **8c** caused complete growth arrest within 48 h, with a more pronounced effect in MT330 cells. Data are presented as mean ± SEM from three independent experiments. Statistical significance was determined at the final time point using one-way ANOVA with post hoc analysis. An Incucyte plot from triplicate wells is shown, which is representative of 3 individual experiments. *p* values *** *p*<0.001 were considered significant.

We next employed the IncuCyte Live-Cell Imaging System to monitor GBM cell proliferation and morphology in real time over a 5-day period. LN229 and MT330 GBM cells were seeded into 96-well plates and treated the following day with compound **8c** at concentrations ranging from 1 to 10 nM. MT330 cells were examined instead of U251 cells because they are a clinically relevant in vivo model of GBM tumorigenesis [30], and U251 cells do not readily form tumors. As shown in Figure 4, vehicle-treated LN229 and MT330 cells rapidly entered a rapid exponential growth phase. However, treatment with 1 nM compound **8c** significantly reduced cell proliferation compared to controls. At 3 and 10 nM, both cell lines exhibited complete growth arrest within 48 h of treatment initiation, with a more pronounced response observed in MT330 cells. Photomicrographs taken during this time revealed morphological changes in both cell lines treated with compound **8c**, suggestive of apoptosis.

### 2.4. Compound ***8c*** Induces the Apoptosis of GBM Cells in Vitro

**Figure 5:**
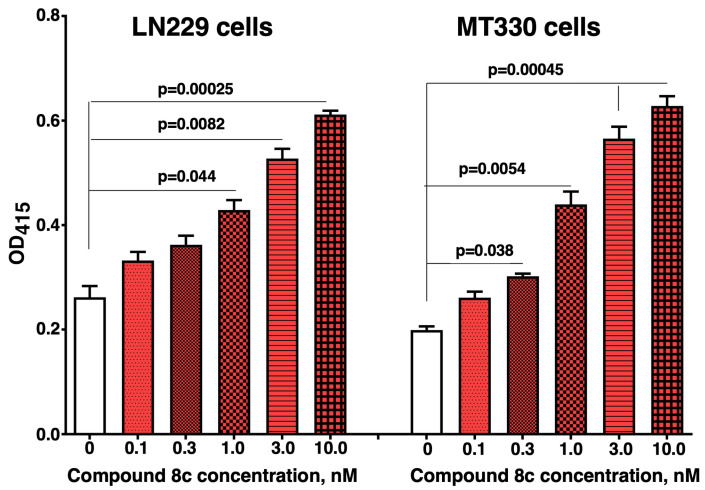
Compound **8c** induces dose-dependent apoptosis in GBM cells. LN229 and MT330 GBM cells were treated with increasing concentrations of compound **8c** for 48 h as indicated. Apoptosis was quantified using a nucleosome-based cell death ELISA, which detects cytoplasmic histone-associated DNA fragments released during apoptotic cell death. The data presented are the means ± SEM from three independent experiments. A significant, dose-dependent increase in apoptosis was observed in both cell lines, with maximal apoptotic activity of compound **8c** detected between 1 and 3 nM. The results from 3 experiments performed in triplicate wells are shown. Statistical significance was determined using one-way ANOVA with post hoc analysis.

We next investigated whether the reduction in GBM cell viability following compound **8c** treatment was attributable in part to the induction of apoptosis. LN229 and MT330 GBM cells were treated with compound **8c** for 48 h, after which apoptosis was assessed using a highly sensitive, cell death ELISA that detects nucleosome release from apoptotic cells, as previously described [30]. As shown in Figure 5, compound **8c** induced apoptosis in LN229 GBM cells in a dose-dependent manner, with significant activity observed at concentrations between 1 and 3 nM, which is consistent with its IC_50_ values from SRB assays. In addition to LN229 cells, we also examined the induction of MT330 GBM cell death by compound **8c**. Similar to the results in LN229 cells, MT330 cells exhibited a robust, dose-dependent apoptotic response to compound **8c**. However, TMZ, which is the standard-of-care chemotherapy for GBM, did not induce apoptosis in either LN229 or MT330 GBM cells even at micromolar concentrations [31], underscoring the distinct and potent pro-apoptotic activity of compound **8c**.

### 2.5. Compound ***8c*** Exhibits Potent Anticancer Activity Against GBM Xenografts in Immunocompromised Mice

Taken together, the data so far have demonstrated that compound **8c** significantly reduces proliferation and induces apoptosis in GBM cells in vitro, suggesting its potential as an anticancer agent. To further evaluate its efficacy, we tested compound **8c** in a GBM xenograft model. MT330 GBM cells were injected subcutaneously into the flanks of immunocompromised mice. Eleven days post-engraftment, mice were randomized into two groups: (a) vehicle control (PEG–saline mixture) and (b) compound **8c**-treated (5 mg/kg, administered intraperitoneally twice weekly). Treatment continued until a humane endpoint was reached in either group.

**Figure 6:**
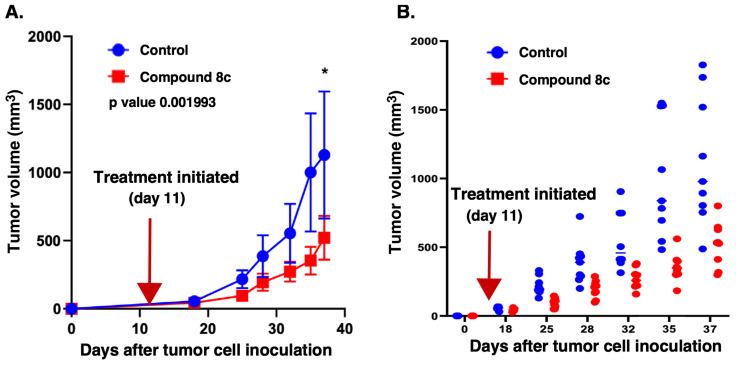
Compound **8c** has potent anticancer activity on GBM xenografts. MT330 cells (5 × 10^5^) were injected into the flanks of 20 female NSG (NOD.Cg-*Prkdc^scid^ Il2rg^tm1Wjl^*/SzJ) immunocompromised mice. At 11 days after tumor injection, palpable tumor masses could be readily discerned. The mice were then randomized into two groups (*N* = 8): a vehicle control group (PEG-saline) and a group of mice treated with compound **8c** at 5 mg/Kg twice weekly. Tumor volumes were measured with calipers on a weekly basis: (**A**) Linear graph of tumor volumes plotted against the number of days after tumor cell inoculation. (**B**) Dot plot of tumor volumes in eight individual mice plotted against the number of days after tumor inoculation. A significant inhibition in tumor growth was observed throughout the course of treatment with compound **8c**. Statistical significance was determined using one-way ANOVA with post hoc analysis. *p* values are indicated as follows: 0.1 (*).

Tumor volumes were measured twice a week using calipers. As shown in Figure 6A, throughout the 25-day time course of treatment with compound **8c**, there was a significant reduction in mean tumor volume compared to controls. Individual tumor volumes throughout the time course of drug treatment are depicted in Figure 6B, which further confirms the markedly reduced growth in the compound **8c**-treated group.

**Figure 7:**
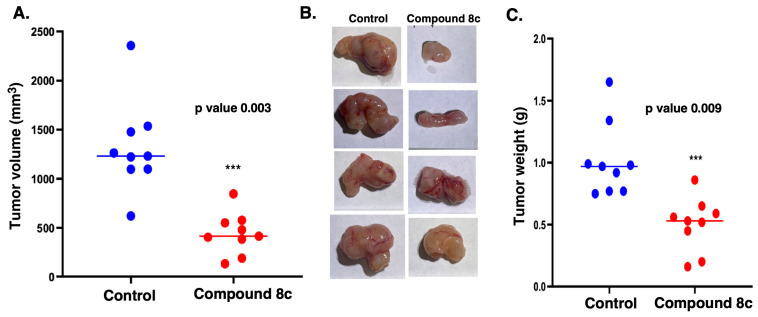
Compound **8c** has a potent effect on GBM xenograft tumor size and weight. Mouse cells were inoculated with MT330 GBM cells and treated with compound **8c** or vehicle, as described in the legend in Figure 6. At 26 days after treatment (37 days after tumor injection), the mice were euthanized and the tumors removed. A significant inhibition in tumor growth was observed at two weeks after treatment with compound **8c**: (**A**) dot plot of tumor volume; (**B**) photograph of three tumors from each group; (**C**) dot plot of tumor weights. Statistical significance was determined using one-way ANOVA with post hoc analysis of 8 tumors from each group. *p* values are indicated as follows: 0.001 (***).

At day 36 post-injection, several mice in the control group reached the humane endpoint (tumor volume equal to or greater than 1000 mm^3^), whereas none of the compound **8c**-treated mice did. At this time point, all mice were euthanized, and tumors were excised, measured, and weighed. Figure 7A shows the dot plots of the tumor volumes in individual mice, showing the trend of smaller tumors in compound **8c**-treated mice. The average tumor size in the compound **8c**-treated group was 441.3 ± 212.2mm^3^, compared to 1322.6 ± 468.8 mm^3^ in the control group—a 66% reduction. Figure 7B visually illustrates the difference in tumor size, with those in compound **8c**-treated mice being substantially smaller than those from control mice. Furthermore, as shown in Figure 7C, the weight of the tumors was markedly lower in compound **8c**-treated mice as compared to control (0.50 ± 0.216 g versus 1.02 ± 0.298 g)

Taken together, the results in Figure 6 and Figure 7 clearly demonstrate that compound **8c** exhibits potent anticancer activity against GBM tumors in vivo.

## 3. Discussion

Gliomas—particularly high-grade variants such as GBM—are among the most aggressive and treatment-resistant tumors of the central nervous system. Despite advances in surgical resection, radiotherapy, and chemotherapy with TMZ, the prognosis for GBM patients remains poor, with median survival rarely exceeding 15 months [26,27,28,29]. A major challenge in treating gliomas is their highly proliferative, invasive, and heterogeneous nature, compounded by the presence of the blood–brain barrier (BBB), which restricts effective drug delivery to tumor sites [27,28,29,30,31].

In recent years, microtubule-targeting agents (MTAs) have emerged as a promising class of chemotherapeutics capable of disrupting cancer cell division, migration, and intracellular transport. These agents act by binding to tubulin—the structural component of microtubules—thereby interfering with microtubule dynamics essential for mitotic spindle formation and cellular integrity [27]. Given the pivotal role of microtubules in glioma cell proliferation and migration, tubulin inhibitors offer potential to address critical therapeutic gaps in glioma treatment. However, translating the success of tubulin inhibitors from other cancer types to glioma therapy has proven difficult. Challenges such as limited BBB penetration, neurotoxicity, and the molecular heterogeneity of glioma cells have hindered clinical efficacy. Several tubulin inhibitors have demonstrated anticancer activity. For instance, Verubulin (MPC-6827, Azixa), a pyrimidine-derived small molecule, inhibits microtubule formation, crosses the BBB, and induces mitotic arrest and apoptosis in various cancer cell lines. Nevertheless, Verubulin failed in phase II clinical trials for recurrent GBM due to insufficient efficacy and unacceptable toxicity [32]. Similarly, BPR0L075 (SCB01A) and BNC105 [33,34] showed preclinical efficacy in multiple cancer models, including GBM, but did not achieve success in clinical trials [29,30,31].

Recent studies have continued to explore colchicine-binding site inhibitors (CBSIs) as viable alternatives for GBM therapy. Liu et al. reported that 2-aryl-4-amide-quinoline derivatives exhibited potent antiproliferative activity and favorable pharmacokinetics in breast cancer models, suggesting broader applicability of strategies [26]. Weng et al. emphasized the structural flexibility and strong cytotoxicity of CBSIs, positioning them as a mainstream approach for overcoming multidrug resistance [27]. In GBM-specific research, Manzoor et al. identified pyrimidine-based CDK6 inhibitors with high binding affinity and stability, reinforcing the therapeutic relevance of pyrimidine scaffolds [28]. Byrne et al. developed pyrazolopyrimidinones with selective cytotoxicity against GBM cells in vitro, further validating nitrogen-containing heterocycles as promising platforms for drug development [29].

Potent compounds were previously developed by our lab, but they raise concerns related to toxicity and metabolic stability. Therefore, we designed a new class of pyrimidinyl dihydroquinoxalinones incorporating various functional groups (hydrogen bond donors and acceptors) at the 2nd position of the pyrimidine ring. These analogs demonstrated strong anticancer activity against multiple cancer cell lines, along with significantly improved metabolic stability [21,22]. Compound **8c** emerged as the lead candidate, demonstrating nanomolar IC_50_ values and superior efficacy compared to TMZ. Structural modifications, particularly secondary amine substitutions and heterocyclic enhancements, were key contributors to its cytotoxic potency.

Live-cell imaging of cells revealed that compound **8c** rapidly inhibited GBM cell proliferation, while apoptosis assays confirmed a robust, dose-dependent induction of cell death at concentrations far lower than those required for TMZ. Importantly, in vivo studies using a GBM xenograft model showed that compound **8c** reduced tumor volume by 66% and tumor weight by over 50%, without apparent toxicity.

Taken together, these findings validate colchicine-site tubulin inhibition as a compelling strategy for GBM treatment and support the continued preclinical development of compound **8c**. Its potent activity, favorable pharmacodynamics, and ability to overcome TMZ resistance position it as a strong candidate for future translational and clinical studies.

## 4. Materials and Methods

### 4.1. Biological Reagents and Cell Cultures

LN229 GBM cell line (ATCC, Manassas, VA, USA) and the MT330 cell line (Department of Neurosurgery, UTHSC) were cultured in DMEM supplemented with 10% fetal bovine serum (HyClone, Logan, UT, USA), 100 IU/mL penicillin, and 100 µg/mL streptomycin. Cells were maintained at 37 °C in a humidified incubator with 5% CO_2_. All cell lines were authenticated by short tandem repeat (STR) analysis.

### 4.2. SRB Assays

LN229 and U251 GBM cells were plated at a density of 5000 to 10,000 cells per well in a 96-well plate and treated with various concentrations of MTAs. After 4 to 5 days of incubation, cells were fixed with trichloroacetic acid, and the plates were washed thoroughly. Sulforhodamine B (SRB) dye was then added to stain the cellular proteins. Following extensive washing to remove unbound dye, the bound SRB was solubilized, and absorbance was measured at 540 nm on a plate spectrophotometer. IC_50_ values were determined using GraphPad Prism 9 software.

### 4.3. Cell Proliferation Assays

For cell proliferation assays, LN229 and MT330 GBM cells were seeded at 2 × 10^3^ cells per well in 96-well plates. After 24 h, plates were treated with or without compound **8c** and placed in the Incucyte Live-Cell Analysis System (Essen Bioscience, Ann Arbor, MI, USA). Cells were incubated at 37 °C, and proliferation was monitored over time by cell confluency using the manufacturer’s software tools.

### 4.4. Cell Death Assays

For cell death assays, LN229 and MT330 GBM cells were seeded at 1 × 10^4^ cells per well in 48-well plates. Following 2 days of drug treatment, apoptosis in the adherent cell population was assessed using the Cell Death Detection ELISA^PLUS^ kit (Roche Diagnostics, Indianapolis, IN, USA), according to the manufacturer’s instructions. This assay quantifies cytoplasmic histone-associated DNA fragments, a hallmark of apoptotic cell death [30].

### 4.5. Mouse Xenograft Experiments

All animal experiments were conducted in accordance with a protocol approved by the Institutional Animal Care and Use Committee (IACUC) at UTHSC (Protocol #17-098). Xenograft tumors were established in five-week-old female NOD.Cg-*Prkdc^scid^ Il2rg^tm1Wjl^*/SzJ immunocompromised mice (Jackson Laboratory, Bar Harbor, ME, USA). For xenograft studies, MT330 cells (5 × 10^5^) were injected subcutaneously into the flanks of mice, as previously described [31]. Once tumors became palpable (usually within 10 to 14 days), mice were randomly assigned to the two treatment groups (9 mice per group) and received intraperitoneal injections of compound **8c** (5 mg/kg body weight) or vehicle (DMSO) twice per week. Tumor volumes were measured weekly using calipers (calculated as width^2^ × length ÷ 2).

## 5. Conclusions

Glioblastoma (GBM) remains one of the most challenging malignancies to treat due to its aggressive nature, resistance to standard therapies, and limited drug penetration across the blood–brain barrier. In this study, we identified and characterized a novel pyrimidine-based tubulin inhibitor, compound **8c**, which targets the colchicine-binding site on tubulin. Compound **8c** demonstrated potent cytotoxicity in vitro across multiple GBM cell lines, outperforming TMZ in both cell viability and apoptosis assays. Live-cell imaging confirmed its ability to induce rapid and complete growth arrest, while ELISA-based assays validated its pro-apoptotic activity at nanomolar concentrations.

Importantly, compound **8c** exhibited strong anticancer efficacy in vivo, significantly reducing tumor volume and weight in a GBM xenograft model without observable toxicity. Future studies should test the efficacy of compound **8c** in intracranial GBM tumor models. If compound **8c** fails to show efficacy on intracranial GBM tumors, this may reflect its inability to cross the blood–brain barrier, which could be addressed by treatment with the other compounds that show in vitro efficacy in Table 1. These results underscore the therapeutic potential of colchicine-site tubulin inhibitors in overcoming TMZ resistance and improving GBM treatment outcomes. The favorable pharmacological profile of compound **8c** supports its continued preclinical development and positions it as a promising candidate for future translational and clinical studies targeting glioblastoma.

## Figures and Tables

**Table 1:** Cytotoxic activities of MTAs on U251 and LN229 GBM cell lines at 72 h after treatment.

ID	IC_50_ (nM) GBM Cell Lines	
	*U251*	*LN229*
Temozolomide	>1000	>1000
**6**	2.49 ± 0.98	1.88 ± 0.67
**8a**	>1000	>1000
**8b**	80.1 ± 6.3	15.6 ± 1.38
**8c**	2.36 ± 0.64	2.09 ± 0.59
**8d**	3.1 ± 1.27	3.15 ± 1.48
**8e**	0.81 ± 0.28	5.82 ± 1.84
**8f**	6.56 ± 2.47	8.36 ± 4.46
**8g**	5.71 ± 3.41	5.66 ± 3.02
**12**	2.09 ± 0.43	2.09 ± −0.43

## Data Availability

The original contributions presented in this study are included in the article/Supplementary Material. Further inquiries can be directed to the corresponding authors.

## Supplementary Materials

The following supporting information can be downloaded at: https://www.mdpi.com/article/10.3390/ph18121891/s1. Experimental Section; The synthesis and characterization of the target compounds; 1H and 13C NMR and HRMS; and HPLC purity results for target compounds.

## Abbreviations

The following abbreviations are used in this manuscript:

GBM	Glioblastoma
TMZ	Temozolomide
MTAs	Microtubule-targeting agents
IC_50_	Half-maximal inhibitory concentration
SRB	Sulforhodamine B
NSG	NOD.Cg-Prkdc*^scid^* *Il2rg^tm1Wj^*^l^/SzJ
