# Supplementary material for: A Pyrimidine-Based Tubulin Inhibitor Shows Potent Anti-Glioblastoma Activity In Vitro and In Vivo
Source: Pharmaceuticals (Basel). 2025 Dec 15;18(12):1891. doi: 10.3390/ph18121891 (PMC13019412; doi:10.3390/ph18121891)
Supplement: Supplementary file 1 [file pharmaceuticals-18-01891-s001.zip › pharmaceuticals-3948005-supplementary.pdf]

A Pyrimidine-Based Tubulin Inhibitor Shows Potent Anti-Glioblastoma Activity In Vitro and In Vivo

Satyanarayana Pochampally [1], Lawrence M. Pfeffer [2,*], Gustavo A. Miranda-Carboni [3], Macey Daniel [3], Jazz I. James [2], Allana Smith [2], Chuan He Yang [2], Hannah R. Kelso [2], Deanna N. Parke [2], Dong-Jin Hwang [1], Wei Li [1] and Duane D. Miller [1,*]

[1] Department of Pharmaceutical Sciences, College of Pharmacy, University of Tennessee Health Science
Center, Memphis, TN 38163, USA; spochamp@uthsc.edu (S.P.); dhwang@uthsc.edu (D.-J.H.); wli@uthsc.edu (W.L.)
[2] Department of Pathology and Laboratory Medicine, Center for Cancer Research, College of Medicine,
University of Tennessee Health Science Center, Memphis, TN 38163, USA; jazijame@uthsc.edu (J.I.J.); asmit611@uthsc.edu (A.S.); cyang@uthsc.edu (C.H.Y.); hanrkels@uthsc.edu (H.R.K.);
dparke2@uthsc.edu (D.N.P.)
[3] Department of Medicine, Center for Cancer Research, College of Medicine, University of Tennessee Health Science Center, Memphis, TN 38163, USA; gmirand1@uthsc.edu (G.A.M.-C.); khd927@uthsc.edu (M.D.)
* Correspondence: lpfeffer@uthsc.edu (L.M.P.); dmiller@uthsc.edu (D.D.M.)

Table of contents:

Experimental Section S2

General Procedure for Synthesis Target Compounds: S3-S8

^{1}H NMR ,^{13}C NMR, HPLC and HRMS spectra's S8-S43

EXPERIMENTAL SECTION

General Methods. All nonaqueous reactions were performed in oven-dried glassware under an inert atmosphere of dry nitrogen. All the reagents and solvents were purchased from Aldrich (St. Louis, MO), Alfa-Aesar (Ward Hill, MA), Combi-Blocks (San Diego, CA), or Ark Pharm (Libertyville, IL) and used without further purification. Analytical thin layer chromatography was performed on silica gel GHLF 10 cm × 20 cm Analtech TLC Uniplates (Analtech, Newark, DE) and visualized by fluorescence quenching under UV light. Silica gel (60 - 120 or 100 - 200 mesh) was used to purify the compounds. ^{1}H NMR and ^{13}C NMR spectra were recorded on a Varian Inova-500 spectrometer (400 MHz) (Agilent Technologies, Santa Clara, CA) or a Bruker Ascend 400 (400 MHz) (Billerica, MA) spectrometer. Chemical shifts are reported in ppm on the δ scale and referenced to the appropriate solvent residual peaks ($CDCl_3$, 7.27 ppm for ^{1}H and 77.23 ppm for ^{13}C; DMSO-d_6, 2.50 ppm for ^{1}H and 39.51 ppm for ^{13}C) and all coupling constants (*J*) are reported in hertz (Hz). Mass spectra were collected on a Bruker amazon SL electrospray/ion trap instrument in the positive and negative modes. High resolution mass spectrometer (HRMS) data were acquired on a Waters Xevo G2-S qTOF (Milford, MA) system equipped with an Acquity I class UPLC system. Porcine brain tubulin (catalog no. T-238P) was purchased from Cytoskeleton, Inc. The purity of all tested compounds was determined to be ≥95% by ^{1}H NMR and HPLC. HPLC was performed on an Agilent 1100 HPLC system (Santa Clara, CA) with a Zorbax SB-C18 column, particle size 3.5 μm, 4.6 mm × 150 mm (Agilent). The mobile phases consist of water with 0.1% formic acid (A) and acetonitrile with 0.1% formic acid (B). A flow rate of 1 mL/min was used. The gradient elution was started at 50% B, reached 100% B from 0 to 9 min, was

maintained at 100% B from 9 to 12 min, was decreased to 50% B from 12 to 15 min and was stopped. Compound purity was monitored with a DAD detector set at 254 nm.

General Procedure for Synthesis Target Compounds:

Synthesis of N-(4-methoxy-2-nitrophenyl)-2-(methylthio)-6,7-dihydro-5H-cyclopenta[d]pyrimidin-4-amine (compound **4***).* A mixture of compound **2** (5 g, 0.05 mol) and 4-methoxy-2-nitroaniline **3** (4.6 g, 0.055 mol) in anhydrous IPA (20 mL) with a catalytic amount of HCl (conc, 5 drops) was stirred at 50 °C for 8 hours and monitored by TLC until the reaction was complete. The reaction mass was diluted with saturated aqueous sodium bicarbonate solution (pH = 7), filtered, washed with water, and dried to obtain compound **4** as an orange solid (7.55 g; 90.3% yield).

*Synthesis of 2-chloro-N-(5-methoxy-2-((2-(methylthio)-6,7-dihydro-5H-cyclopenta[d]pyrimidin-4-yl)amino)phenyl)acetamide (*compound **5***).* A mixture of compound **4** (7.5 g, 0.045 mol) and zinc powder (3.0 g, 0.09 mol) in 60 mL of dichloromethane (CH_2Cl_2) in the presence of 1 mL of acetic acid was stirred at 0 °C for 0.5 hour. This mixture was filtered through a celite bed, and the filtrate was concentrated to obtain the aniline derivative (compound **4a**), which was immediately dissolved in acetone (50 mL), powered potassium carbonate (10.5 g, 0.15 mol) was added, and the mixture was cooled to 0 °C. Chloroacetyl chloride (3 mL, excess) was dropped slowly into the mixture, which was stirred at 0 °C for another 2 hours. The mixture was diluted with water, extracted with CH_2Cl_2, washed with brine solution, dried over sodium sulfate (Na_2SO_4), and concentrated. The crude product was purified by column chromatography to obtain compound **5** as a bright pink solid (6.2 g; 63.8% yield).

*Synthesis of 7-methoxy-4-(2-(methylthio)-6,7-dihydro-5H-cyclopenta[d]pyrimidin-4-yl)-3,4-dihydroquinoxalin-2(1H)-one (*compound **6***).* Compound **5** (5.0 g, 0.026 mol) was dissolved in anhydrous THF (50 mL), and a 60% sodium hydride (0.7 g, 0.039 mol) solution was

added portion wise at 0 °C and slowly allowed to warm to room temperature while stirring until the reaction was complete, as determined by TLC monitoring. The mixture was poured into ice-water, and the solid product, compound **6,** was removed by filtration, washed with water, and dried to obtain an ash colored solid (3.0 g; 66.6% yield); ^{1}H NMR (400 MHz, $CDCl_3$) δ 9.62 (s, 1H), 7.34 (d, *J* = 9.1 Hz, 2H), 6.78 (dd, *J* = 8.8, 2.8 Hz, 1H), 4.25 (s, 2H), 3.83 (s, 3H), 2.94 (t, *J* = 7.8 Hz, 2H), 2.73 (s, 2H), 2.39 (s, 3H), 2.15 (dd, *J* = 17.0, 9.6 Hz, 2H); ^{13}C NMR (100 MHz, $CDCl_3$) δ 168.40, 156.73, 131.17, 122.96, 115.74 ,108.16, 102.36, 55.78, 49.64, 30.99, 22.60, 14.21; HRMS [C17H18N4O2S+] calculated 343.1229, found 343.1233; HPLC purity 95.8%; melting point (Mp) = 170 – 171 °C.

*Synthesis of 7-methoxy-4-(2-(methylsulfonyl)-6,7-dihydro-5H-cyclopenta[d]pyrimidin-4-yl)-3,4-dihydroquinoxalin-2(1H) one (compound **7**).* A mixture of compound **6** (2.5 g, 0.014 mol) and potassium peroxymonosulfate (6.1 g, 0.073 mol) in water/methanol (1:1 v/v) was stirred at room temperature for 5 hours and then the reaction mixture was diluted with water, filtered and dried under vacuum to produce compound **7** without further purification (2.5 g; 92.6% yield).

*Synthesis of 4-(2-amino-6,7-dihydro-5H-cyclopenta[d]pyrimidin-4-yl)-7-methoxy-3,4-dihydroquinoxalin-2(1H)-one (compound **8a**).* A mixture of compound **7** (100 mg, 0.26 mmol) and NaN_3 (excess) in 1,4-dioxane (5 mL) was heated to 90 °C for 8 hours in open air condition. The mixture was poured into ice-water, the solid was collected by filtration, washed with water, and dried. The crude product was purified by column chromatography to afford pure compound **8a** as a light brown solid (68 mg; 83.8% yield); ^{1}H NMR (400 MHz, DMSO-d_6) δ 10.61 (s, 1H), 6.76 (d, *J* = 8.4 Hz, 1H), 6.59 – 6.54 (m, 2H), 6.23 (s, 2H), 4.34 (s, 2H), 3.72 (s, 3H), 2.58 (t, *J* = 7.6 Hz, 2H), 2.08 – 2.04 (m, 2H), 1.79 - 1.75 (m, 2H); ^{13}C NMR (100 MHz, DMSO-d_6) δ 167.78, 157.83, 156.73, 156.30, 155.58, 150.40, 133.05, 122.97, 120.44, 119.08, 107.36, 102.30, 55.85, 50.51, 32.35, 29.74, 23.09, 21.22, 14.54;

HRMS [C16H18N5O2+] calculated 312.1460, found 312.1467; HPLC purity 98.2%; Mp = 208 - 209 °C.

*Synthesis of 7-methoxy-4-(2-methoxy-6,7-dihydro-5H-cyclopenta[d]pyrimidin-4-yl)-3,4-dihydroquinoxalin-2(1H)-one (compound **8b**).* A mixture of compound **7** (100 mg, 0.29 mmol) and 0.5*M* sodium methoxide in MeOH (5 mL) was heated to 90 °C for 6 hours in a sealed tube. The mixture was poured into ice-water, the solid was collected by filtration, washed with water, and dried. The crude product was purified by column chromatography to afford pure compound **8b** as a blood red solid (75 mg; 88.7% yield); ^{1}H NMR (400 MHz, DMSO-d_6) δ 10.66 (s, 8H), 6.86 (d, *J* = 8.3 Hz, 7H), 6.58 (t, *J* = 5.6 Hz, 2H), 4.44 (s, 2H), 3.86 (s, 3H), 3.73 (s, 3H), 2.71 (t, *J* = 7.6 Hz, 2H), 2.14 (t, *J* = 7.1 Hz, 2H), 1.86 (dd, *J* = 14.6, 7.2 Hz, 2H); ^{13}C NMR (100 MHz, DMSO-d_6) δ 177.04, 168.20, 164.33, 158.09, 157.07, 132.88, 123.14, 121.00, 113.21, 107.41, 102.09, 55.78, 54.77, 49.57, 33.79, 30.51, 22.68; HRMS [C17H19N4O3+] calculated 327.1457, found 327.1459; HPLC purity 98.1%; Mp = 163 - 164 °C.

*Synthesis of 7-methoxy-4-(2-(methylamino)-6,7-dihydro-5H-cyclopenta[d]pyrimidin-4-yl)-3,4-dihydroquinoxalin-2(1H)-one (compound **8c**).* A mixture of compound **7** (100 mg, 0.26 mmol) and 2*M* methyl amine in tetrahydrofuran (0.4 mL, 8 mmol) in 1,4-dioxane was heated to 110 °C for 7 hours in a sealed tube. The mixture was poured into ice-water, the solid was collected by filtration, washed with water, and dried. The crude product was purified by column chromatography to afford pure compound **8c** as an off white solid (69 mg; 80.0% yield); ^{1}H NMR (400 MHz, DMSO-d_6) δ 10.58 (s, 1H), 6.75 (d, *J* = 8.4 Hz, 1H), 6.65 (q, *J* = 4.7 Hz, 1H), 6.56 (dd, *J* = 12.2, 2.7 Hz, 2H), 4.36 (s, 2H), 3.71 (s, 3H), 2.77 (d, *J* = 4.8 Hz, 3H), 2.59 (t, *J* = 7.4 Hz, 2H), 2.06 (t, *J* = 7.1 Hz, 2H), 1.78 – 1.75 (m, 2H); ^{13}C NMR (100 MHz, DMSO-d_6) δ 176.36, 168.53, 162.94, 157.29, 156.30, 132.43, 122.39, 122.11, 107.32, 102.05, 55.72, 49.40, 30.40, 28.49, 22.54; HRMS [C17H20N5O2+] calculated 326.1617, found 326.1624; HPLC purity 96.7%; Mp = 201 - 202 °C.

*Synthesis of 4-(2-(ethylamino)-6,7-dihydro-5H-cyclopenta[d]pyrimidin-4-yl)-7-methoxy-3,4-dihydroquinoxalin-2(1H)-one (compound **8d**).* A mixture of compound **7** (100 mg, 0.26 mmol) and 2*M* ethyl amine in tetrahydrofuran (0.4 mL, 8 mmol) in 1,4-dioxane was heated to 110 °C for 7 hours in a sealed tube. The mixture was poured into ice-water, the solid was collected by filtration, washed with water, and dried. The crude product was purified by column chromatography to afford pure compound **8d** as an off white solid (69 mg; 80.0% yield); ^{1}H NMR (400 MHz, DMSO-d_6) δ 10.60 (s, 1H), 6.77 (d, *J* = 8.3 Hz, 1H), 6.71 (t, *J* = 5.2 Hz, 1H), 6.57 (d, *J* = 5.5 Hz, 2H), 4.35 (s, 2H), 3.72 (s, 3H), 3.27 (m, 2H), 2.59 (t, *J* = 7.55 Hz, 2H), 2.07 (m, 2H), 1.78 (m, 2H), 1.11 (t, *J* = 7.1 Hz, 3H); ^{13}C NMR (100 MHz, DMSO-d_6) δ 176.36, 168.52, 162.29, 157.32, 156.30, 132.43, 122.41, 122.12, 107.31, 102.04, 67.46, 55.71, 49.42, 35.95, 34.14, 30.42, 25.58, 22.52, 15.46; HRMS [C17H20N5O2+] calculated 340.1773, found 340.1769; HPLC purity 96.9%; Mp = 250 - 252 °C.

*Synthesis of 4-(2-(cyclopropylamino)-6,7-dihydro-5H-cyclopenta[d]pyrimidin-4-yl)-7-methoxy-3,4-dihydroquinoxalin-2(1H)-one (compound **8e**).* A mixture of compound **7** (100 mg, 0.26 mmol) and cyclopropyl amine (45 mg, 0.8 mmol) in 1,4-dioxane was heated to 80 °C for 10 hours in a sealed tube. The mixture was poured into ice-water, the solid was collected by filtration, washed with water, and dried. The crude product was purified by column chromatography to afford pure compound **8e** as a pale yellow solid (60 mg; 60% yield); ^{1}H NMR (400 MHz, DMSO-d_6) δ 10.60 (bs, 1H), 6.95 (d, *J* = 3.3 Hz, 1H), 6.79 (d, *J* = 8.5 Hz, 1H), 6.59 - 6.55 (m, 2H), 4.38 (s, 2H), 3.75 (s, 3H), 2.69 (dq, *J* = 10.6, 3.6 Hz, 1H), 2.61 (t, *J* = 7.5 Hz, 2H), 2.08 (t, *J* = 7.1 Hz, 2H), 1.79 (dd, *J* = 14.5, 7.3 Hz, 2H), 0.62 (dt, *J* = 6.5, 3.1 Hz, 2H), 0.44 - 0.43 (m, 2H); ^{13}C NMR (100 MHz, DMSO-d_6) δ 176.31, 168.54, 163.27, 157.23, 156.35, 132.49, 122.50, 122.08, 107.31, 102.03, 66.79, 55.72, 49.43, 34.12, 30.45, 24.38, 22.55, 6.89; HRMS [C19H22N5O2+] calculated 352.1773, found 352.1778; HPLC purity 98.2%; Mp = 171 - 172 °C.

*Synthesis of 4-(2-(1H-imidazol-1-yl)-6,7-dihydro-5H-cyclopenta[d]pyrimidin-4-yl)-7-methoxy-3,4-dihydroquinoxalin-2(1H)-one (compound **8f**).* A mixture of compound **7** (100 mg, 0.29 mmol), imidazole (54 mg, 0.8 mmol) and diisopropanolamine (DIPA) (71 mg, 0.5 mmol) in 1,4-dioxane was heated to 110 °C for 12 hours in a sealed tube. The mixture was poured into ice-water, the solid was collected by filtration, washed with water, and dried. The crude product was purified by column chromatography to afford pure compound **8f** as a light yellow solid (76 mg; 80% yield); ^{1}H NMR (400 MHz, DMSO-d_6) δ 10.69 (s, 1H), 8.54 (s, 1H), 7.92 (s, 1H), 7.08 (s, 1H), 6.95 (d, J = 8.3 Hz, 1H), 6.61 (d, J = 9.1 Hz, 2H), 4.55 (s, 2H), 3.74 (s, 3H), 2.83 (t, J = 7.6 Hz, 2H), 2.23 (t, J = 7.1 Hz, 2H), 1.92 - 1.88 (m, 2H); ^{13}C NMR (100 MHz, DMSO-d_6) δ 177.38 (s), 168.13, 157.62, 157.28, 153.13, 136.08, 133.09, 130.34, 123.25, 120.74, 117.57, 117.22, 107.30, 102.11, 66.79, 55.79, 49.74, 34.12, 31.00, 22.52; HRMS [C19H19N6O2+] calculated 363.1569, found 363.1577; HPLC purity 99.1%; Mp = 242 - 243 °C.

*Synthesis of 4-(2-isothiocyanato-6,7-dihydro-5H-cyclopenta[d]pyrimidin-4-yl)-7-methoxy-3,4-dihydroquinoxalin-2(1H)-one (compound **8g**).* A 0.5*M* solution of thiophosgene (0.55 mL, 0.4 mmol) in anhydrous CH_2Cl_2 was cooled to 0 °C under argon and a solution of compound **8a** (100 mg, 0.3 mmol) in anhydrous CH_2Cl_2 and diisopropanolamine (0.7 mL) was added. The resulting solution was allowed to warm to room temperature over 8 hours. The reaction mass was quenched with 1*N* HCl (5 mL) and extracted with CH_2Cl_2 (20 mL x 2). The combined organic layers were dried over sodium sulfate and concentrated. The crude product was purified by column chromatography to afford a pale-yellow isothiocyanate solid (50 mg; 44.0% yield); ^{1}H NMR (400 MHz, DMSO-d_6) δ 10.72 (s, 1H), 6.91 (d, J = 8.5 Hz, 1H), 6.60 (d, J = 7.8 Hz, 2H), 4.43 (s, 2H), 3.74 (s, 3H), 2.78 (t, J = 7.6 Hz, 2H), 2.20 (t, J = 7.2 Hz, 2H), 1.90 - 1.86 (m, 2H); ^{13}C NMR (100 MHz, DMSO-d_6) δ 182.66, 166.12, 156.44, 154.36, 153.29, 153.26, 127.11, 126.48, 119.80, 116.90, 109.81, 55.90, 55.54, 34.45, 26.89, 22.32; HRMS [C17H16N5O2S+] calculated 354.1025, found 354.1031; HPLC purity 95.8%; decomposes at 139 °C.

*Synthesis of 7-methoxy-4-(2-methyl-6,7-dihydro-5H-cyclopenta[d]pyrimidin-4-yl)-3,4-dihydroquinoxalin-2(1H)-one (compound **12**).* Compound **11** (100 mg, 0.026 mol) was dissolved in anhydrous THF (2.5 mL), and a 60% sodium hydride (20 mg) solution was added portion wise at 0 °C and slowly allowed to warm to room temperature while stirring until the reaction was complete, as determined by TLC monitoring. The mixture was poured into ice-water, and the solid product, compound **12,** was removed by filtration, washed with water, and dried to obtain an off white solid (60 mg; 75% yield); ^{1}H NMR (400 MHz, DMSO-d_6) δ 10.66 (s, 1H), 6.79 (d, J = 9.2 Hz, 1H), 6.58 (s, 2H), 4.43(s, 2H), 3.73 (s, 3H), 2.74 (t, J = 7.3 Hz, 2H), 2.47 (s, 3H), 2.17 (t, J =6.8 Hz, 2H), 1.92−1.74 (m, 2H); ^{13}C NMR (100 MHz, DMSO-d_6) δ 175.62, 168.29, 165.56, 156.81, 156.70, 132.72, 122.31, 121.55, 116.82, 107.26, 102.12, 55.38, 49.57, 33.99, 30.90, 25.78, 22.36; HRMS [C17H19N4O2+] calculated 311.1508; found 311.1507; HPLC purity 97.5%; Mp 227−229 °C.

Spectra's of ^{1}H- NMR, ^{13}C-NMR, HPLC and HRMS:

^{1}H-NMR of compound 6:

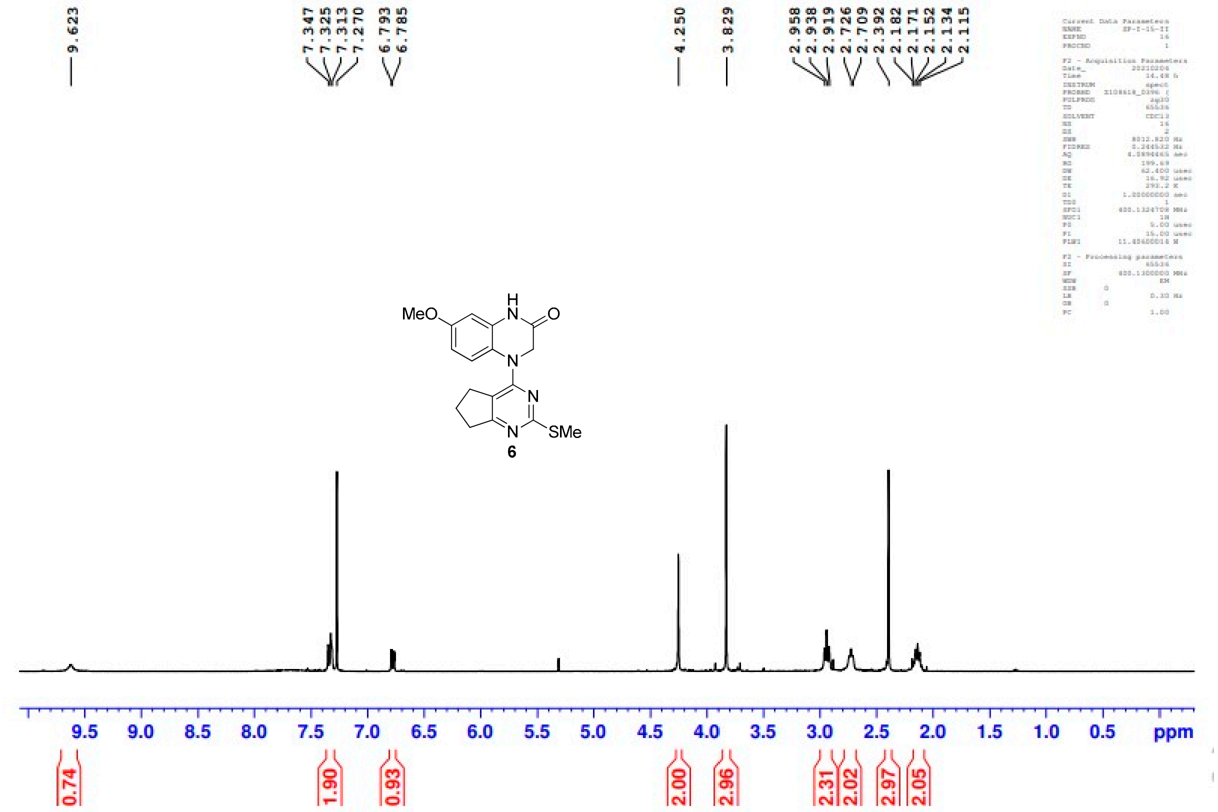

^{13}C-NMR of compound 6:

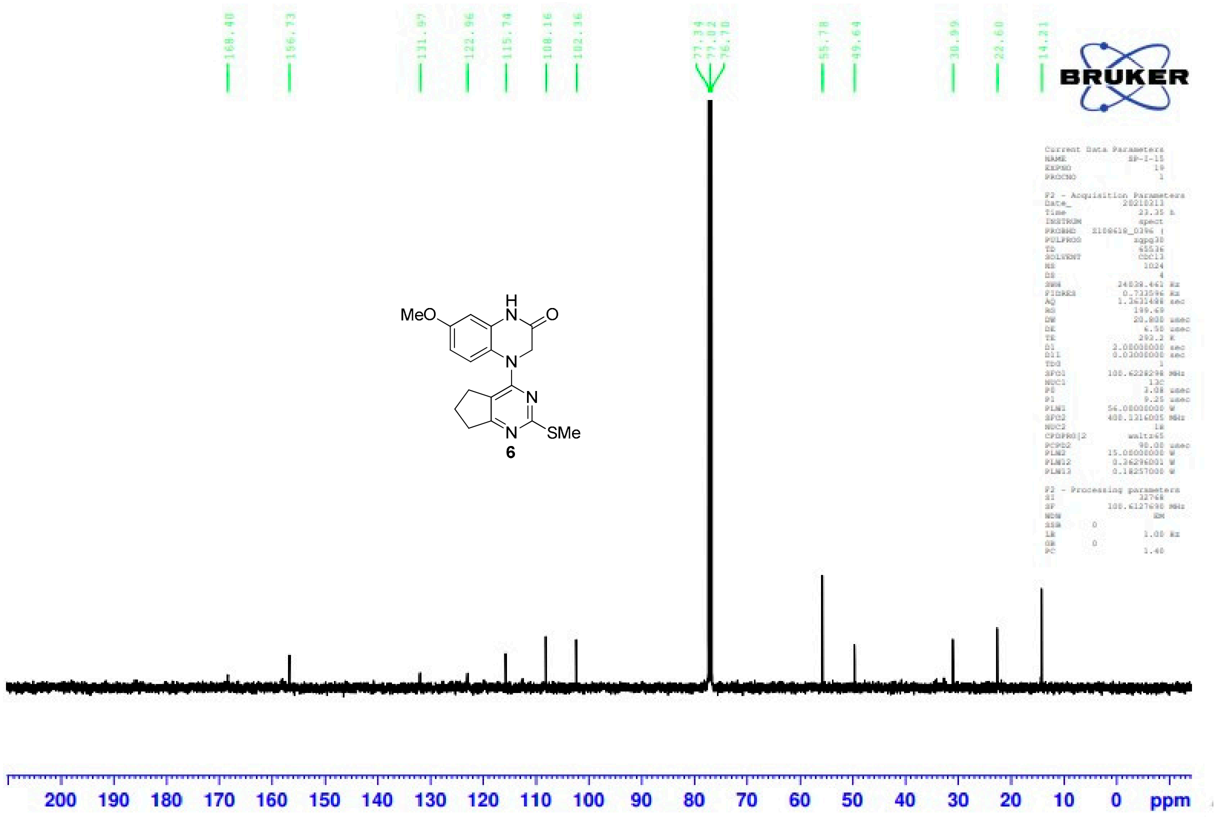

HPLC of compound 6:

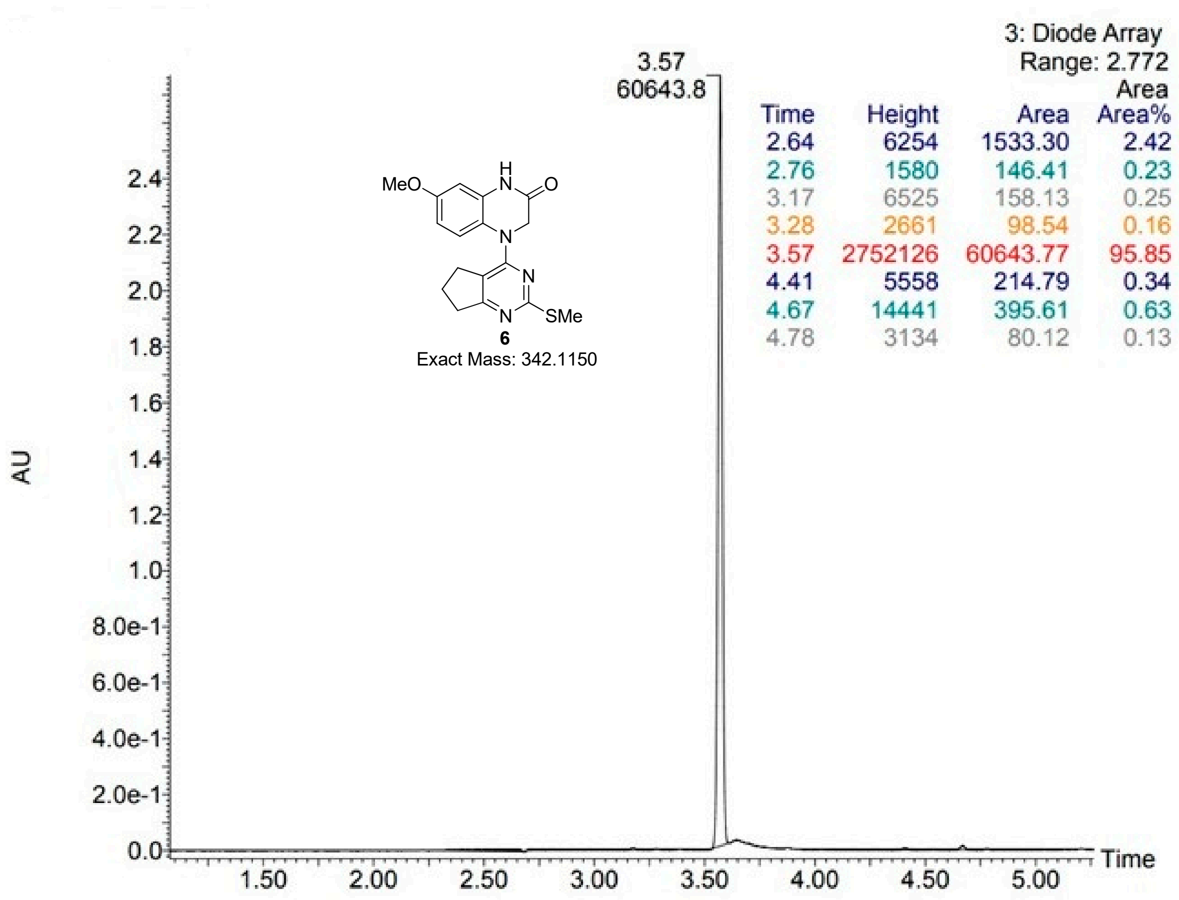

HRMS of compound 6:

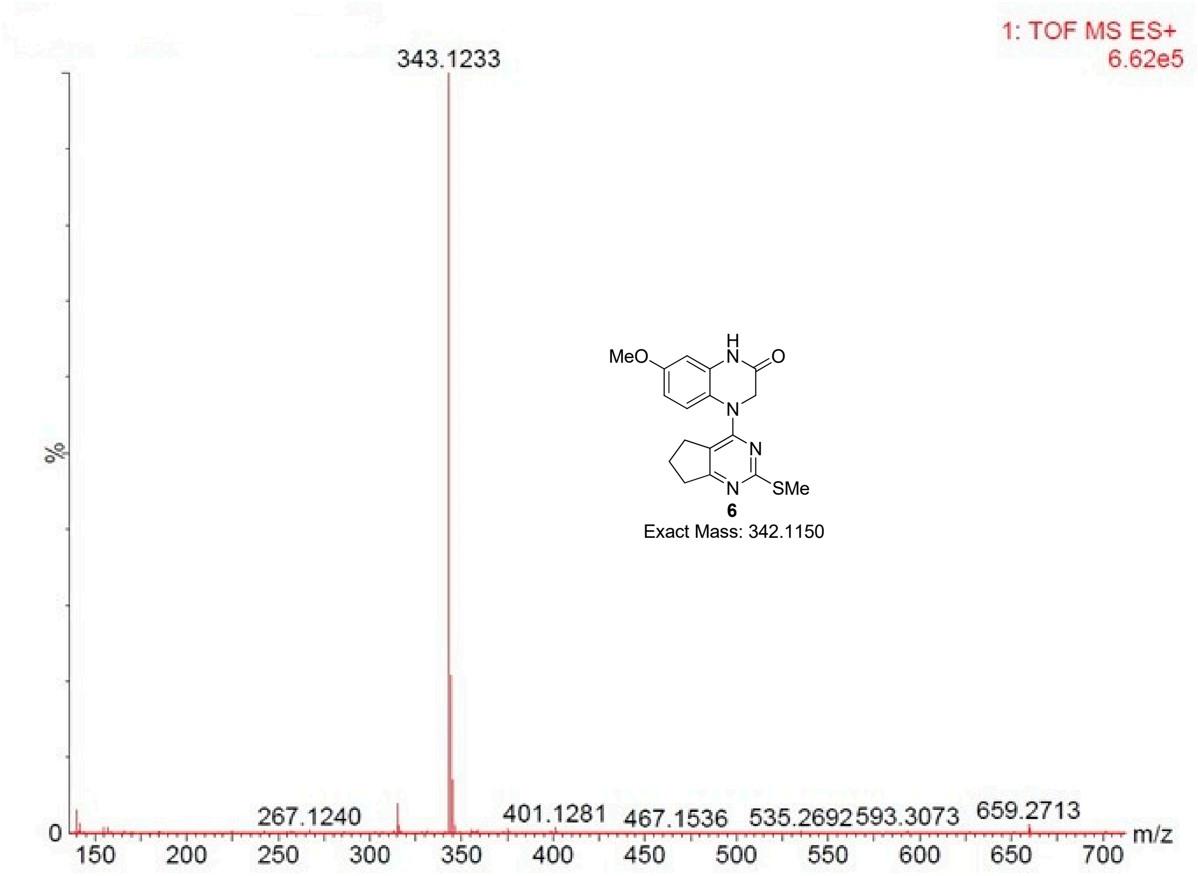

^{1}H-NMR of compound 8a:

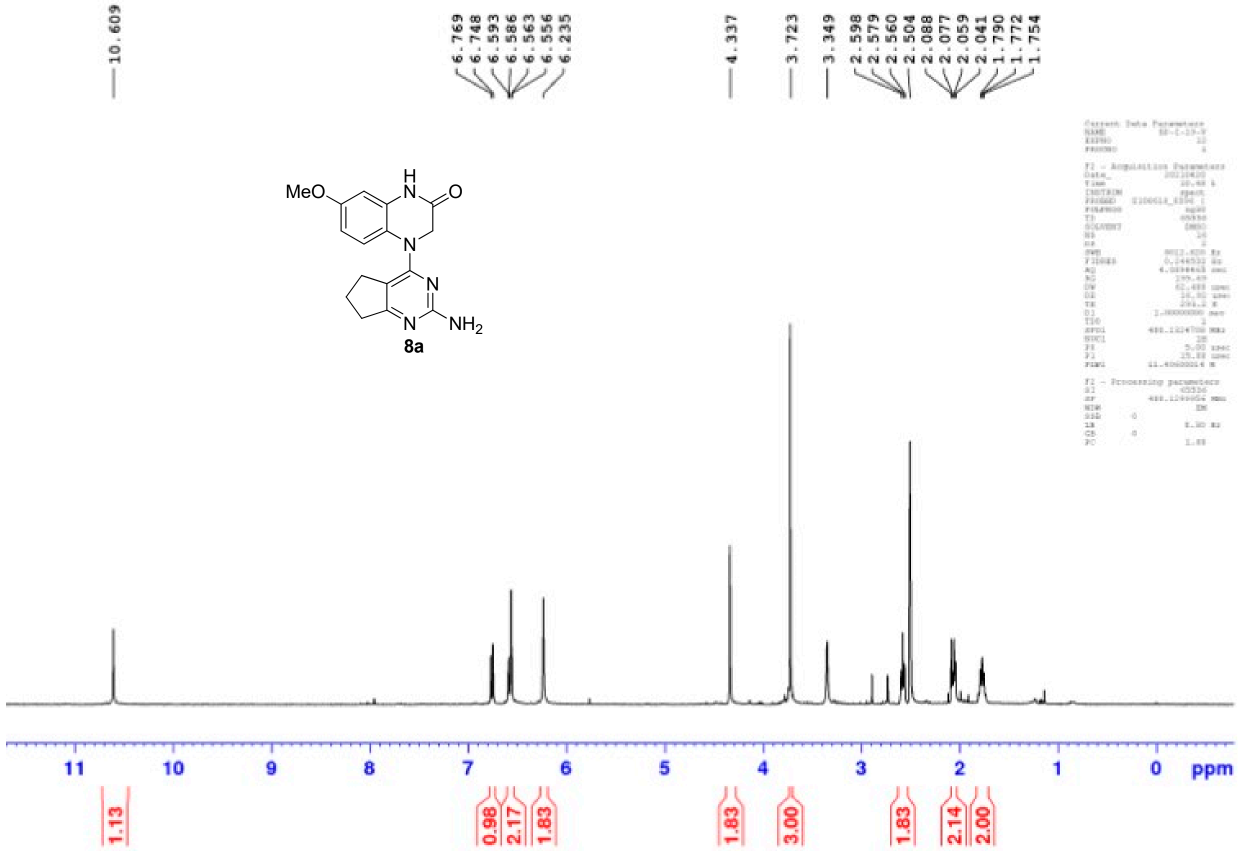

^{13}C-NMR of compound 8a:

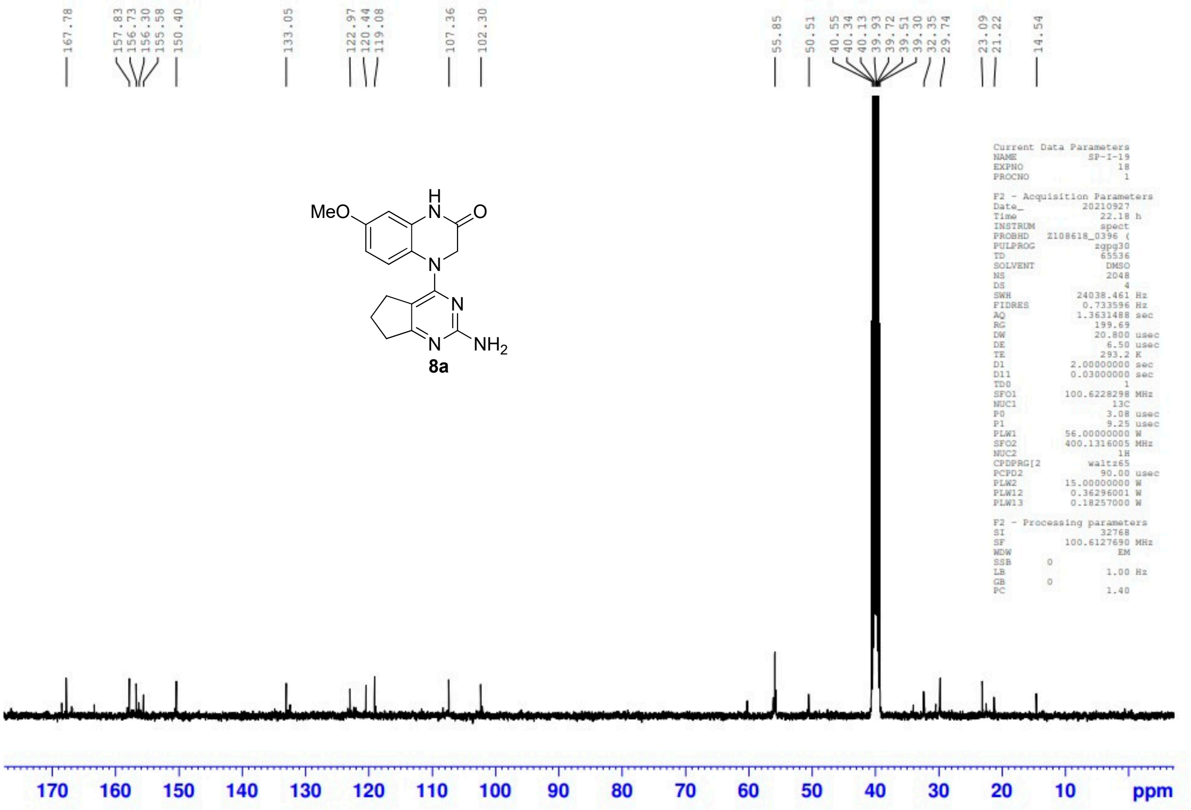

HPLC of compound 8a:

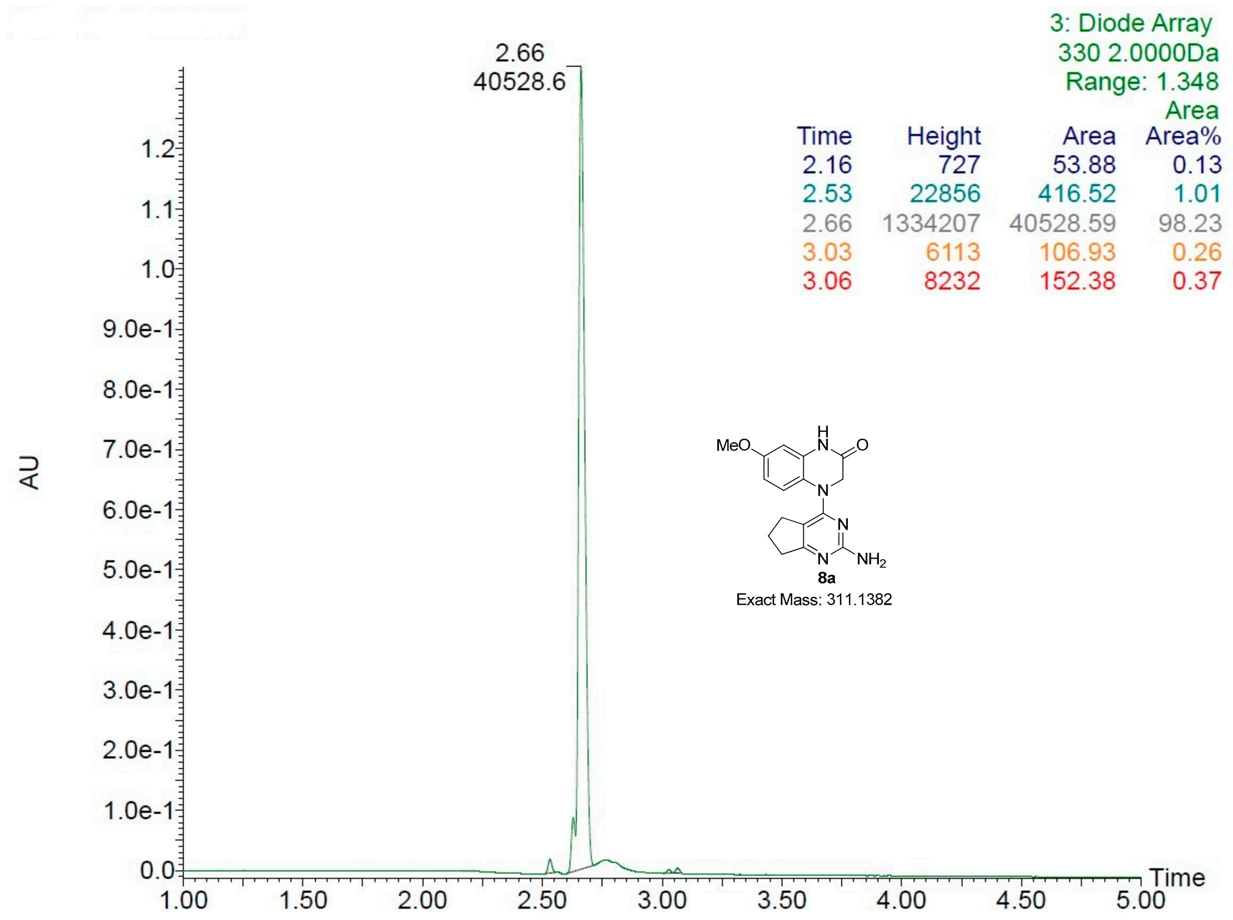

HRMS of compound 8a:

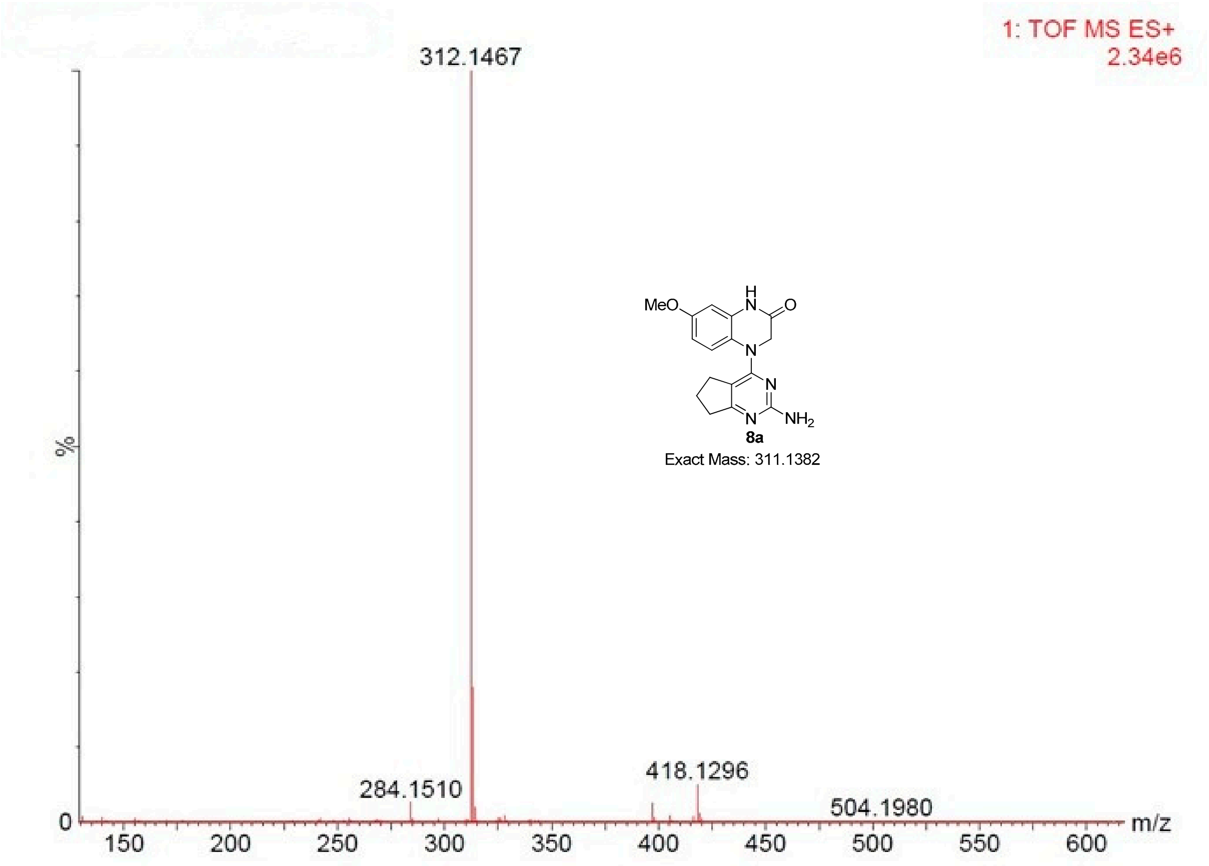

^{1}H-NMR of compound 8b:

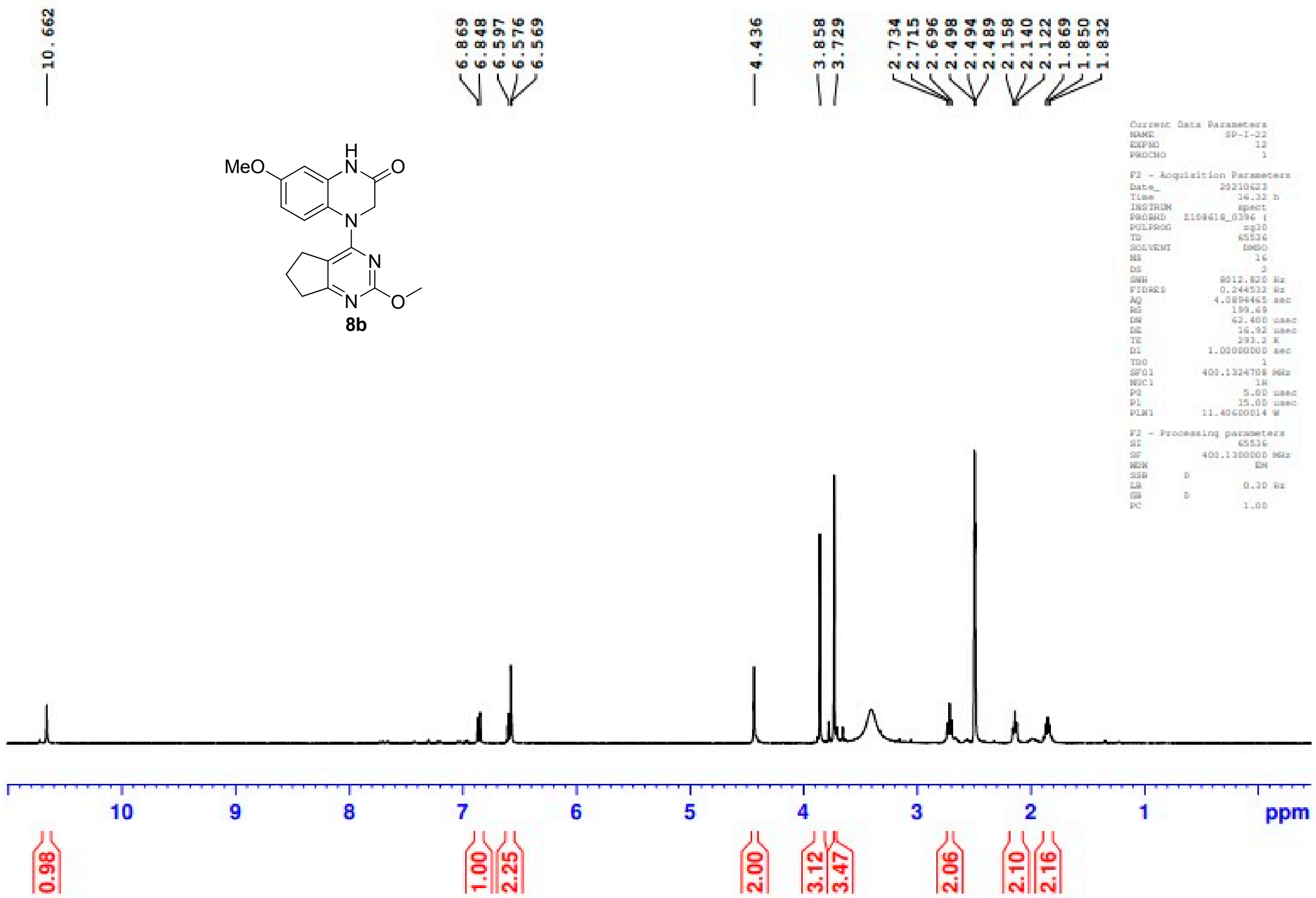

^{13}C-NMR of compound 8b:

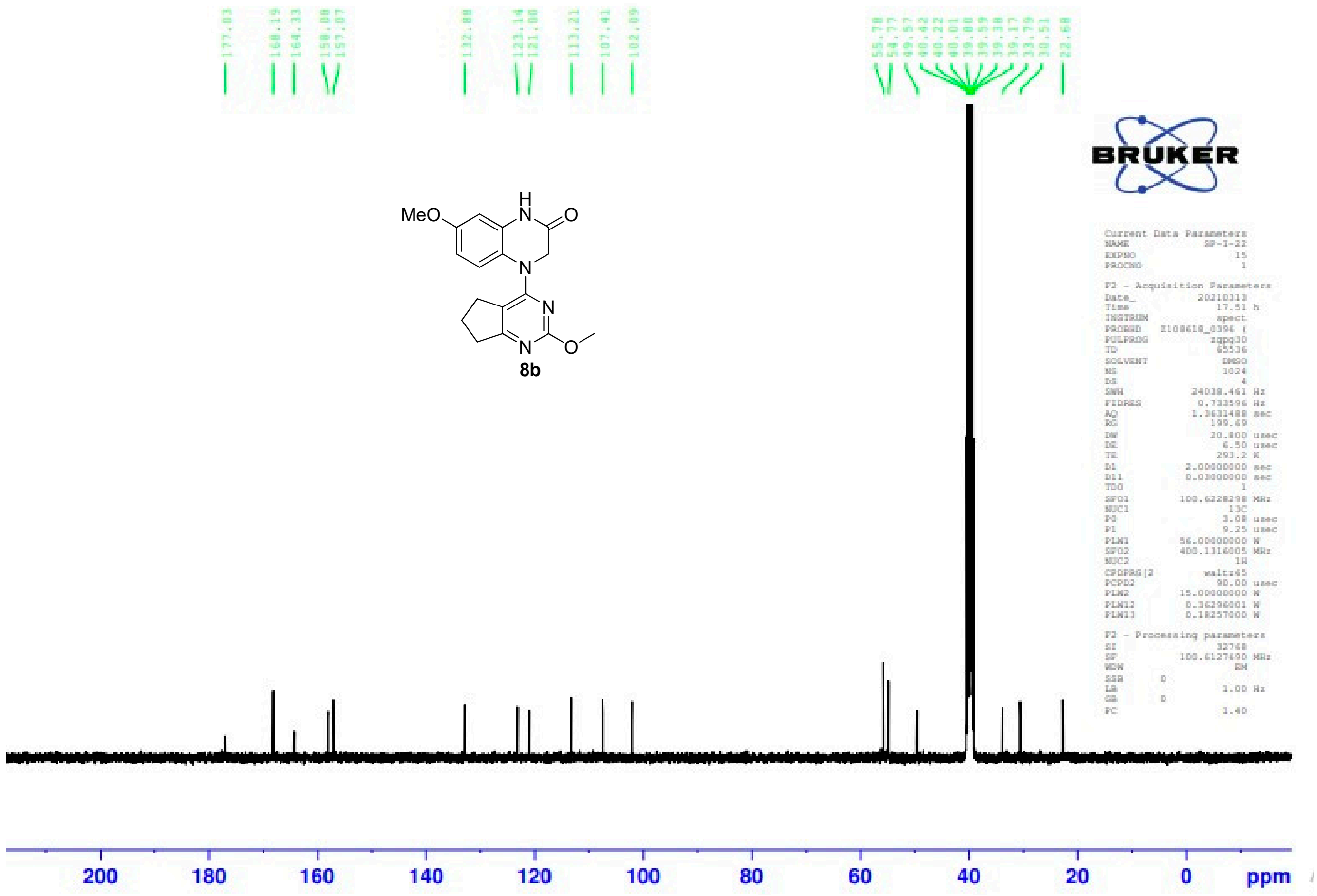

HPLC of compound 8b:

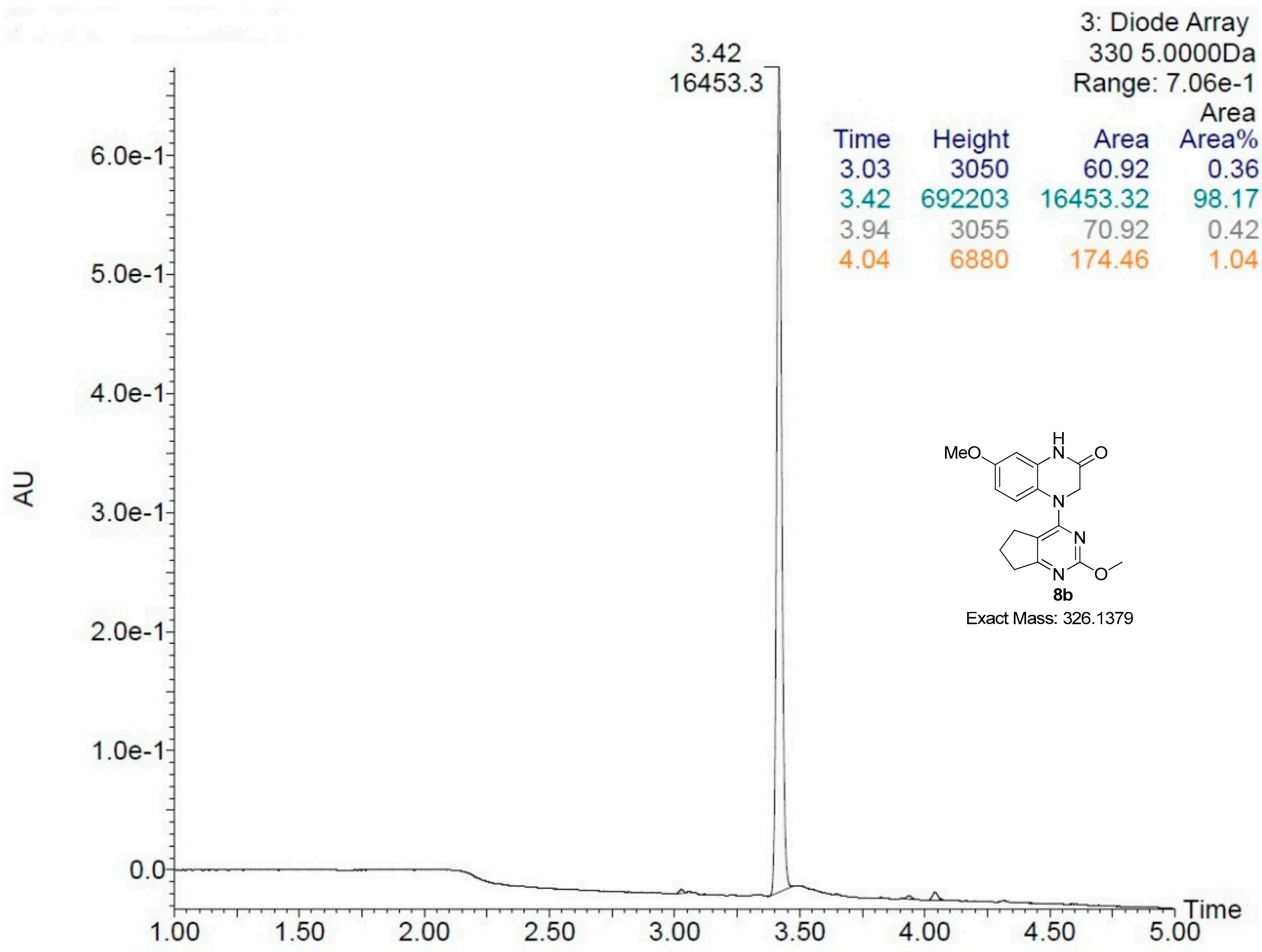

HRMS of compound 8b:

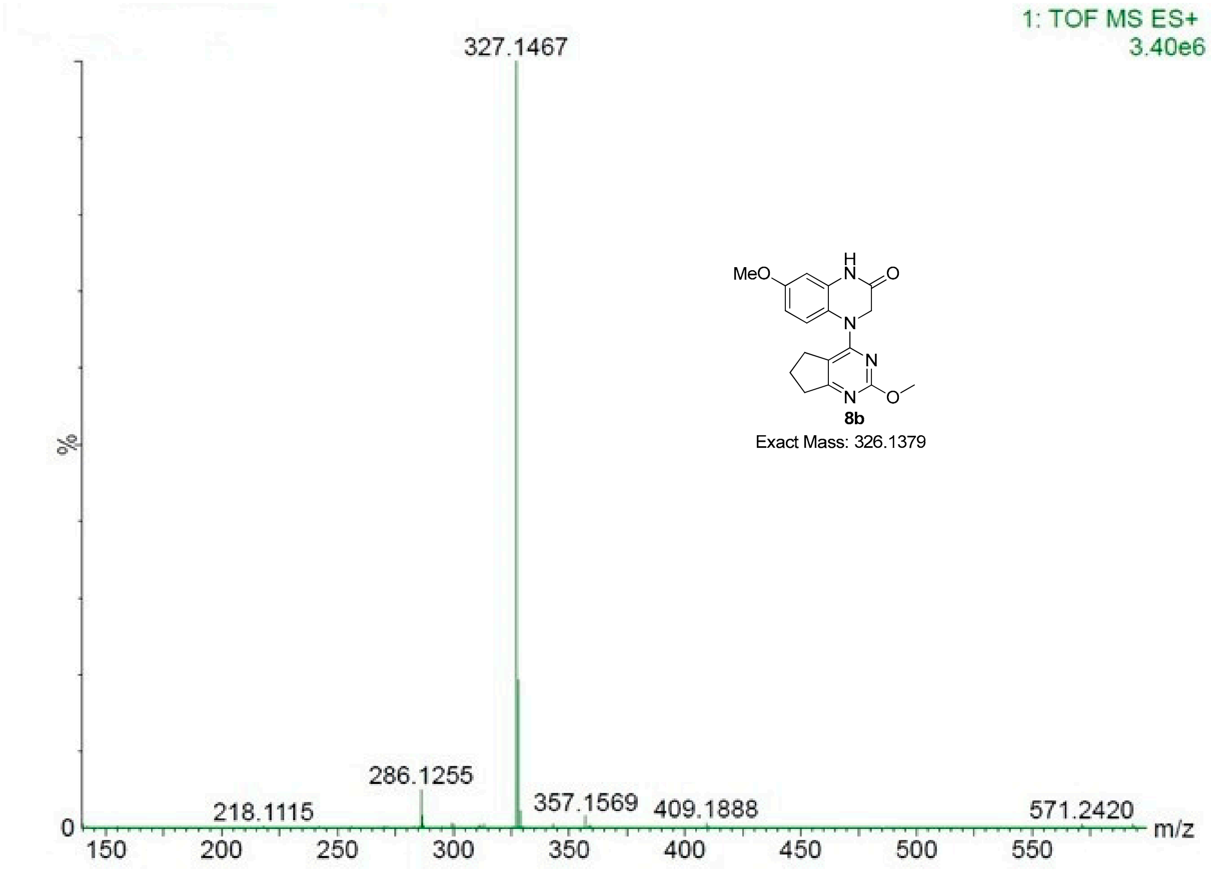

^{1}H-NMR of compound 8c:

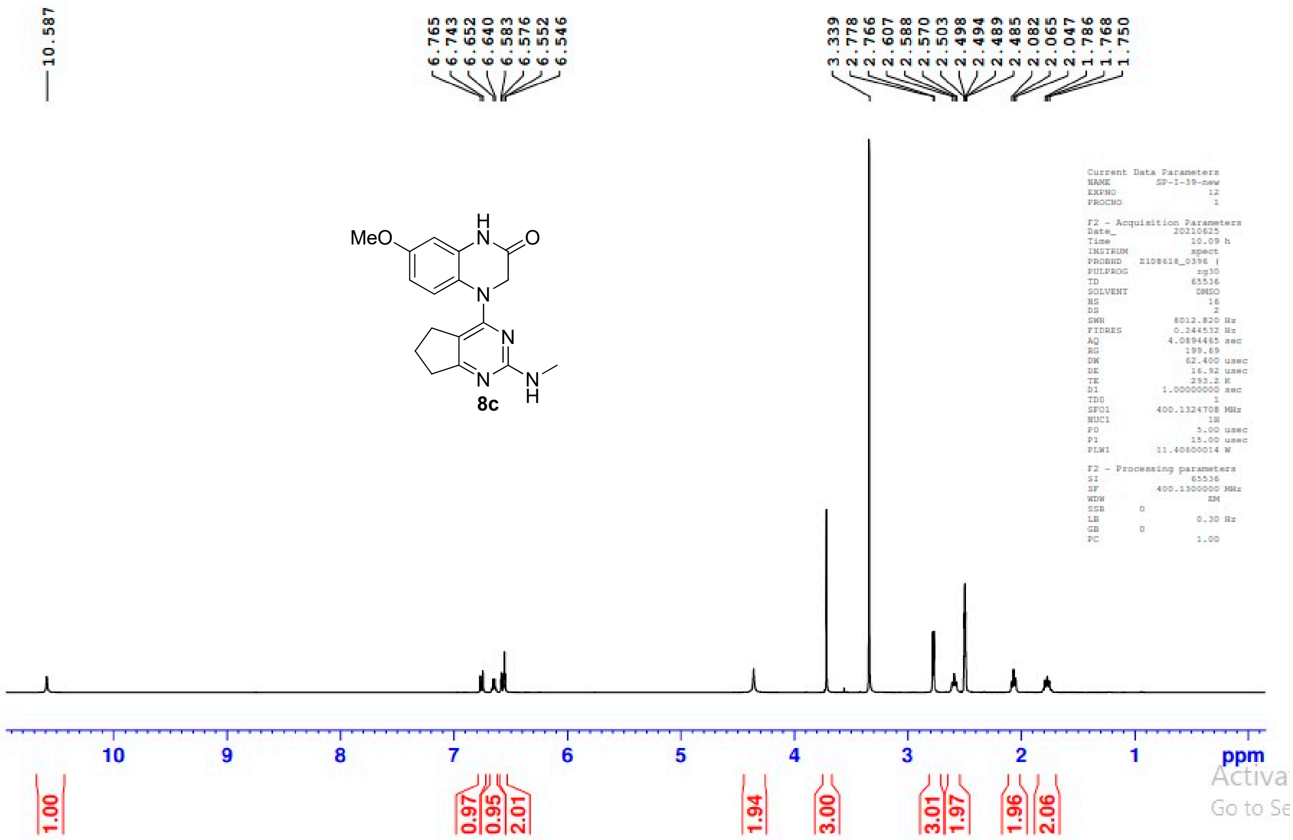

^{13}C-NMR of compound 8c:

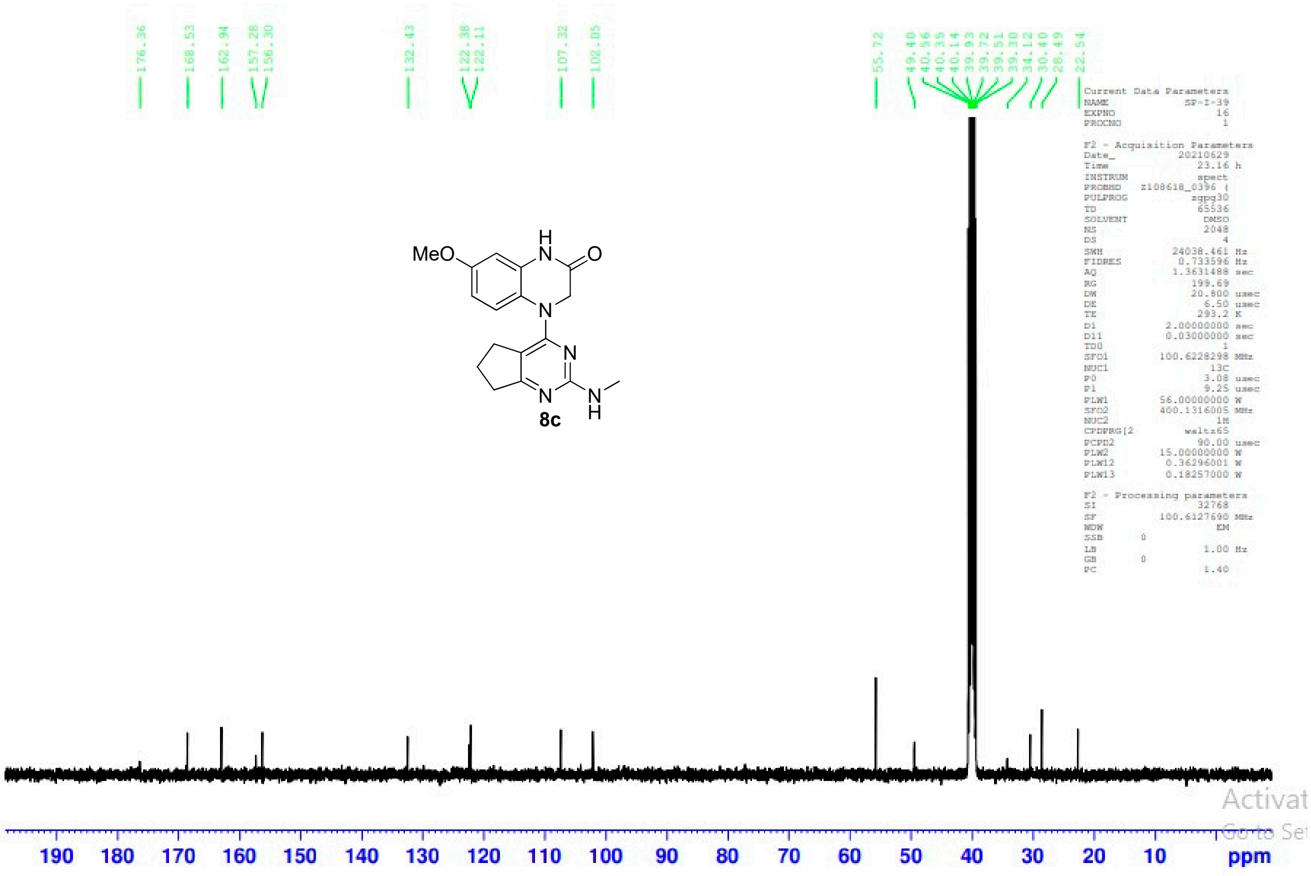

HPLC of compound 8c:

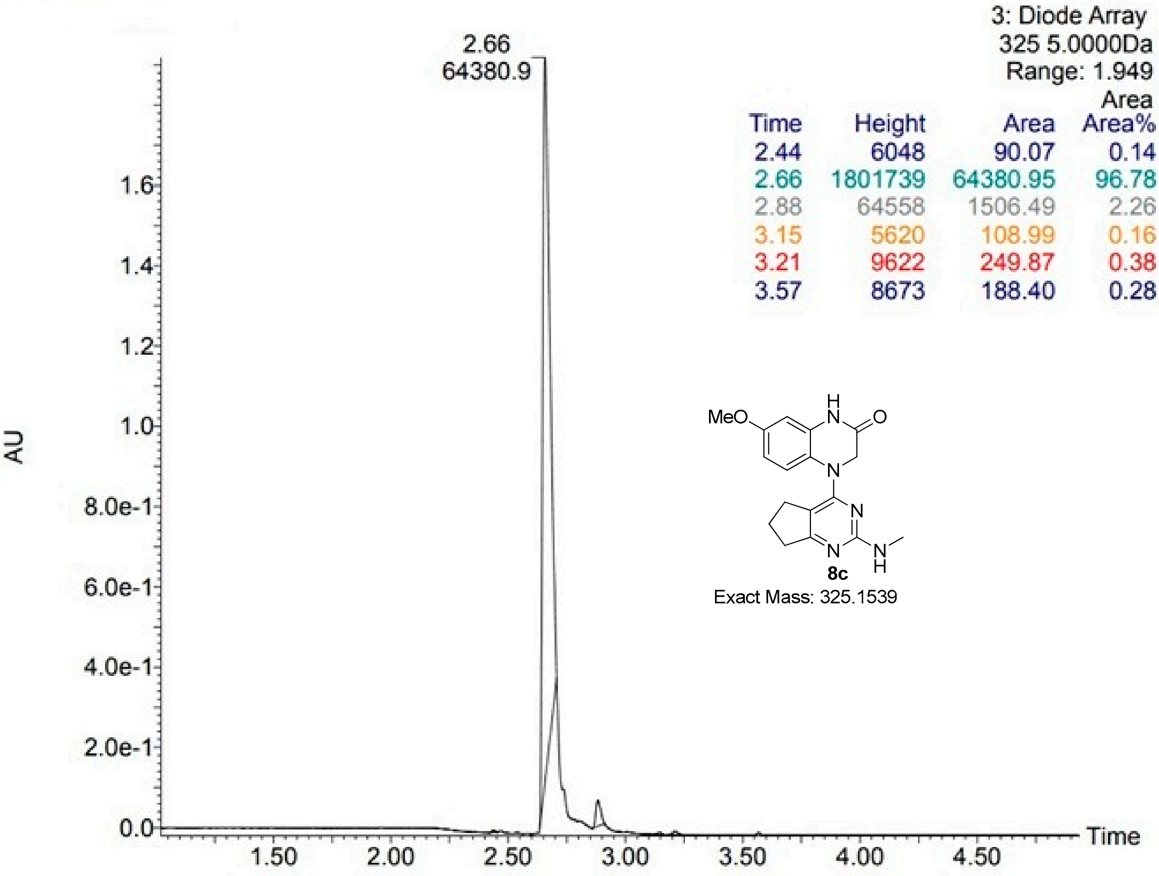

Time	Height	Area	Area Area%
2.44	6048	90.07	0.14
2.66	1801739	64380.95	96.78
2.88	64558	1506.49	2.26
3.15	5620	108.99	0.16
3.21	9622	249.87	0.38
3.57	8673	188.40	0.28

HRMS of compound 8c:

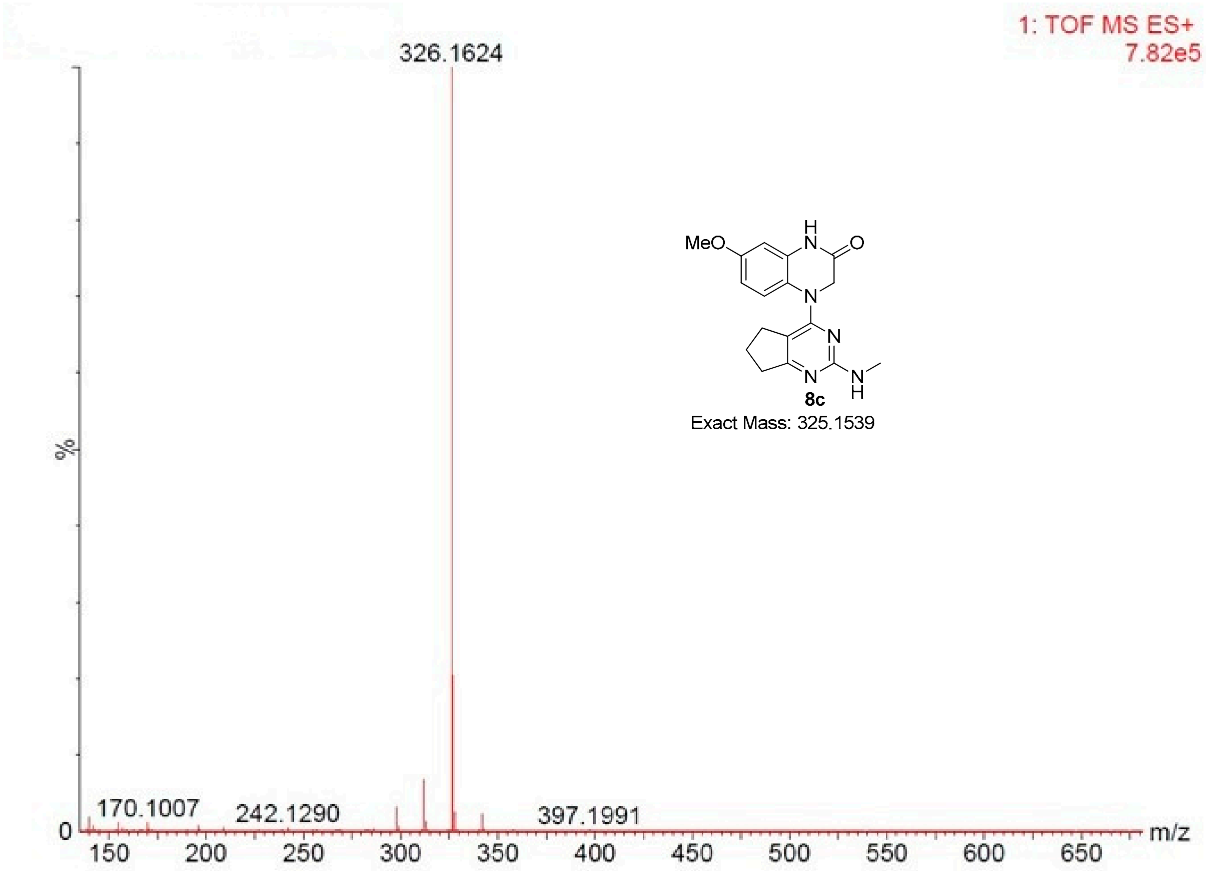

^{1}H-NMR of compound 8d:

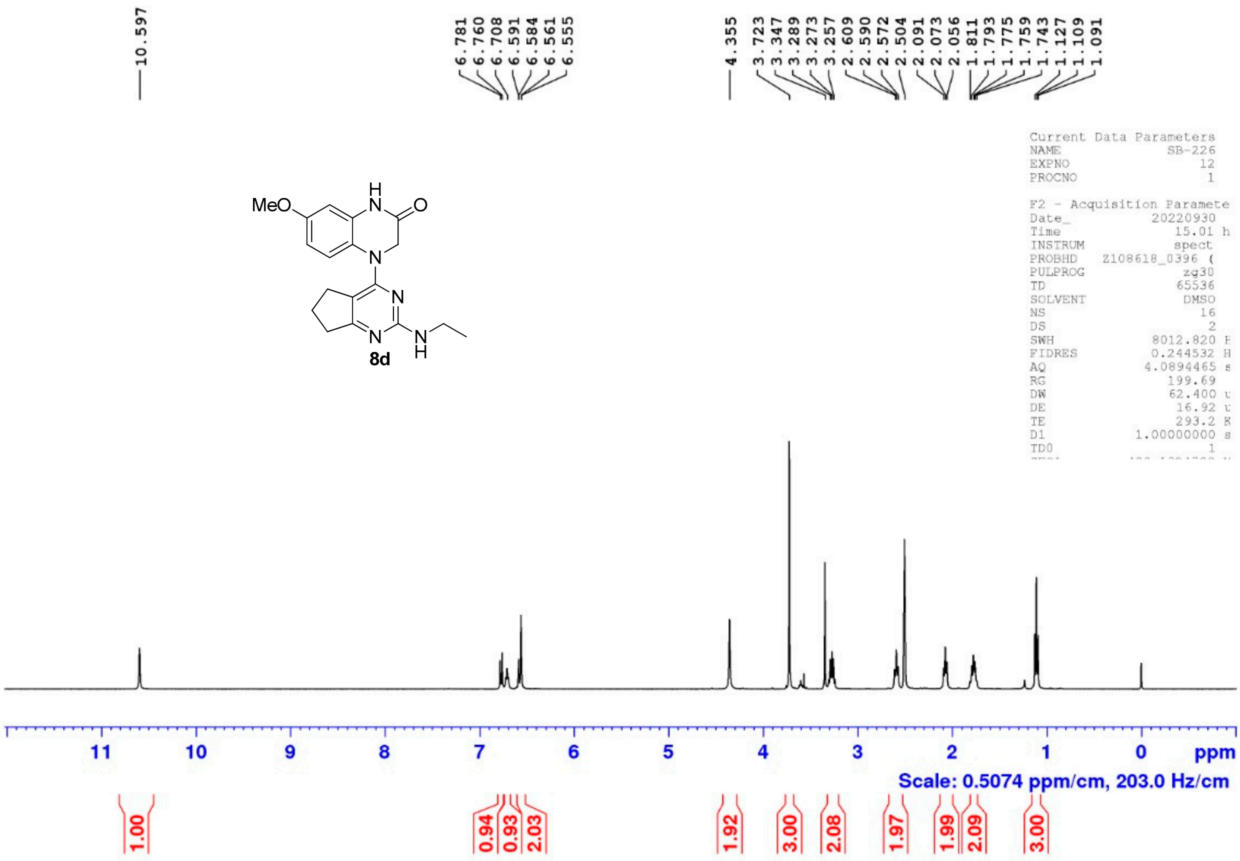

^{13}C-NMR of compound 8d:

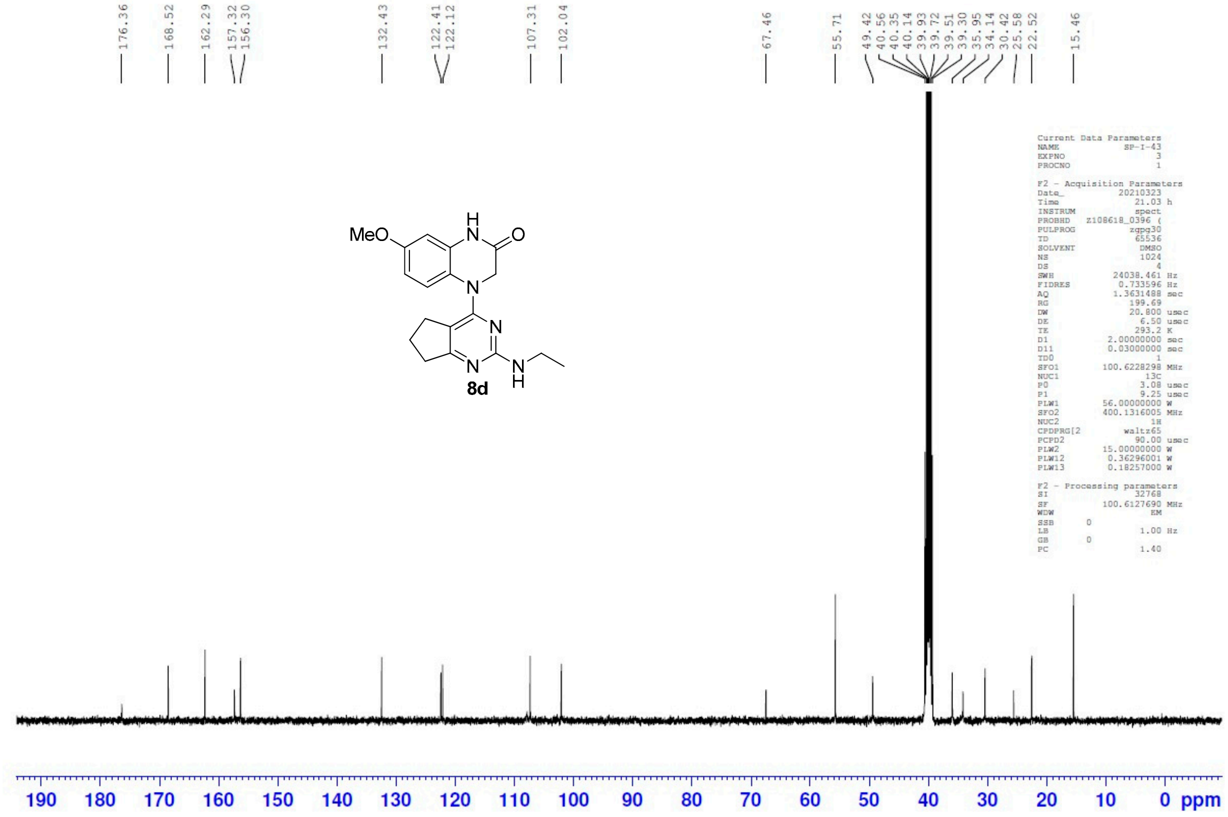

HPLC of compound 8d:

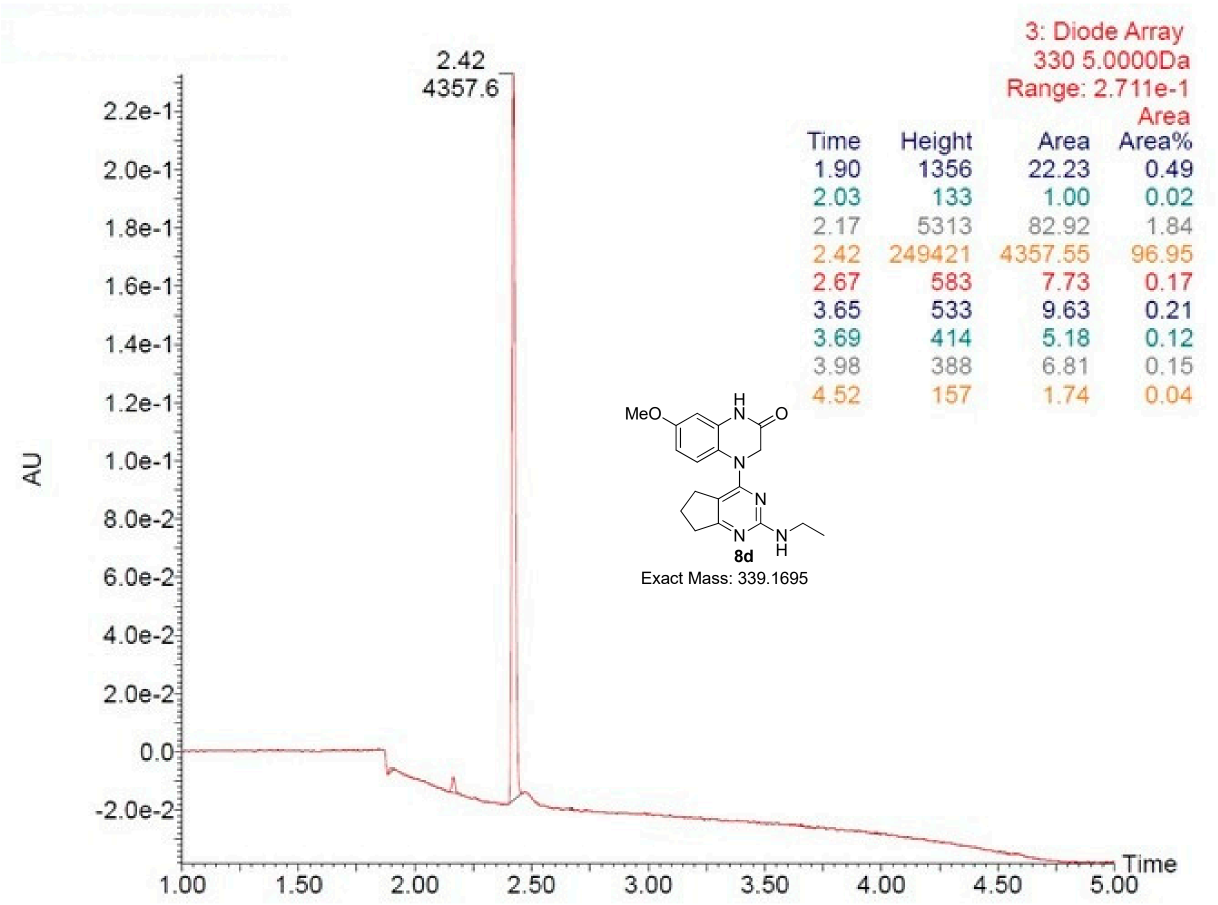

HRMS of compound 8d:

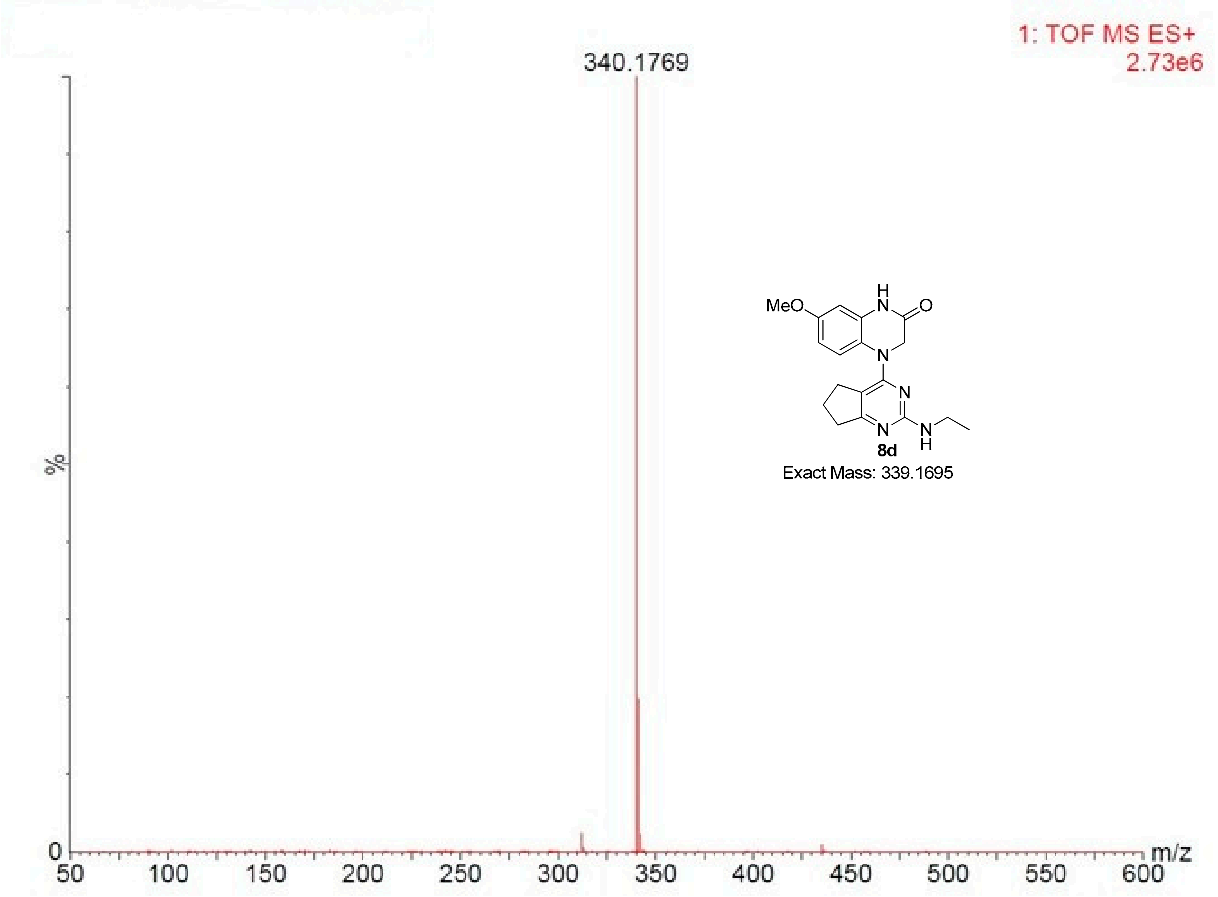

^{1}H-NMR of compound 8e:

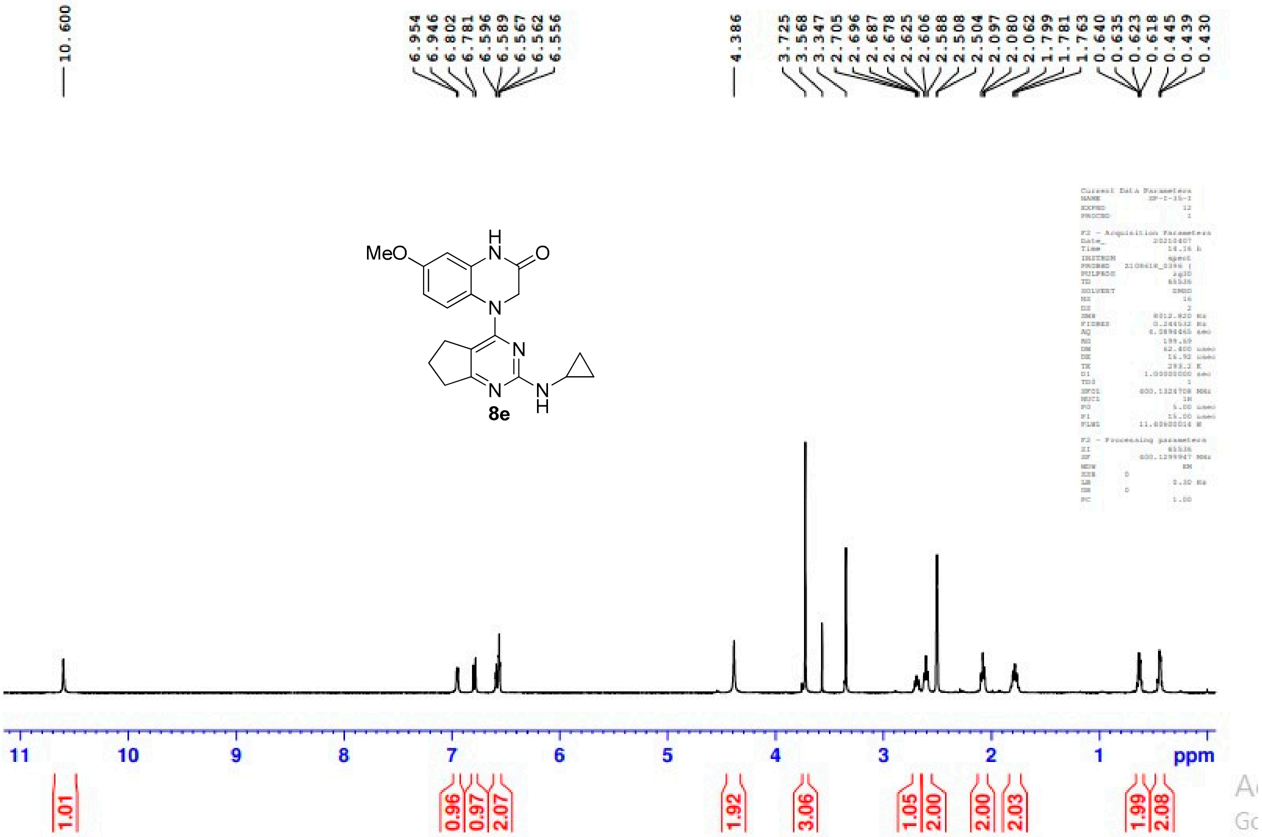

^{13}C-NMR of compound 8e:

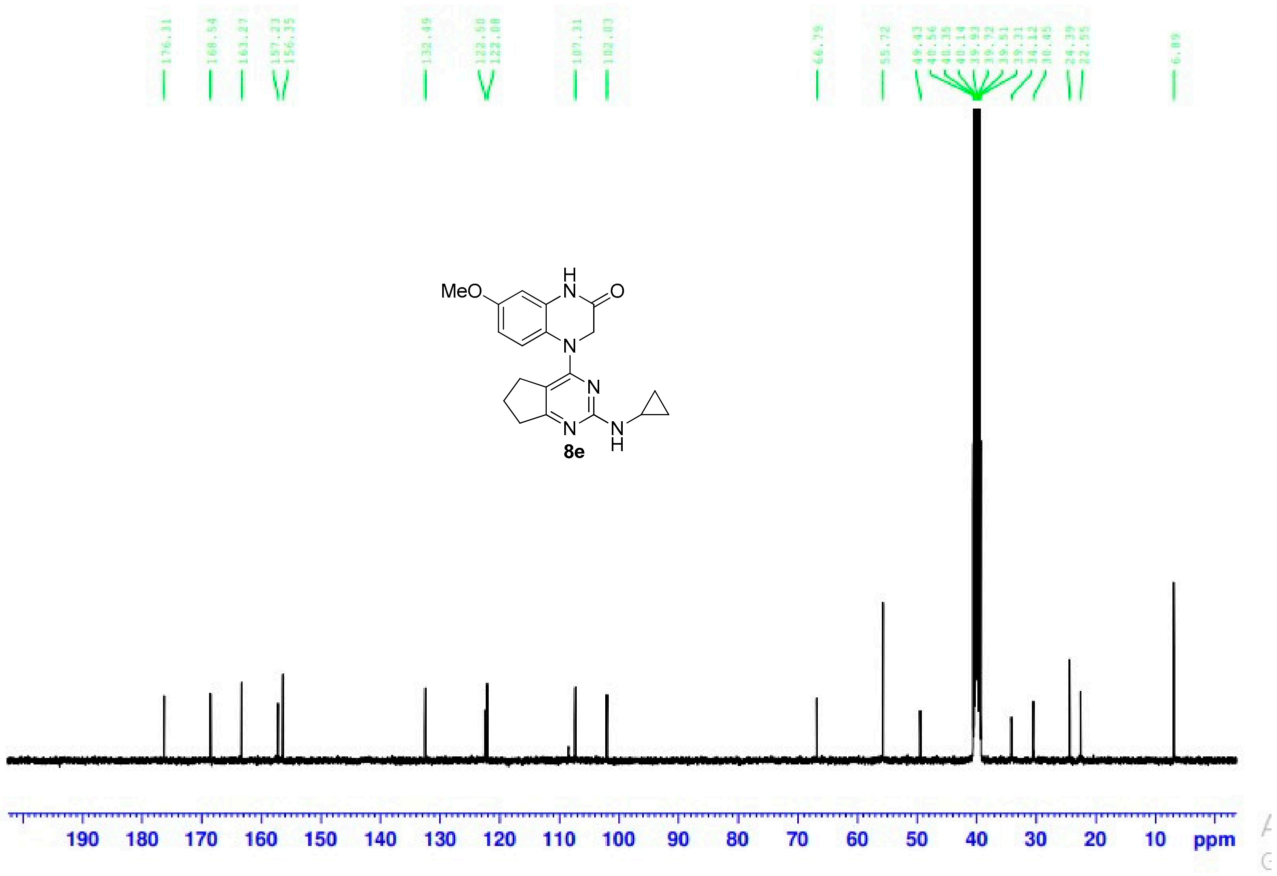

HPLC of compound 8e:

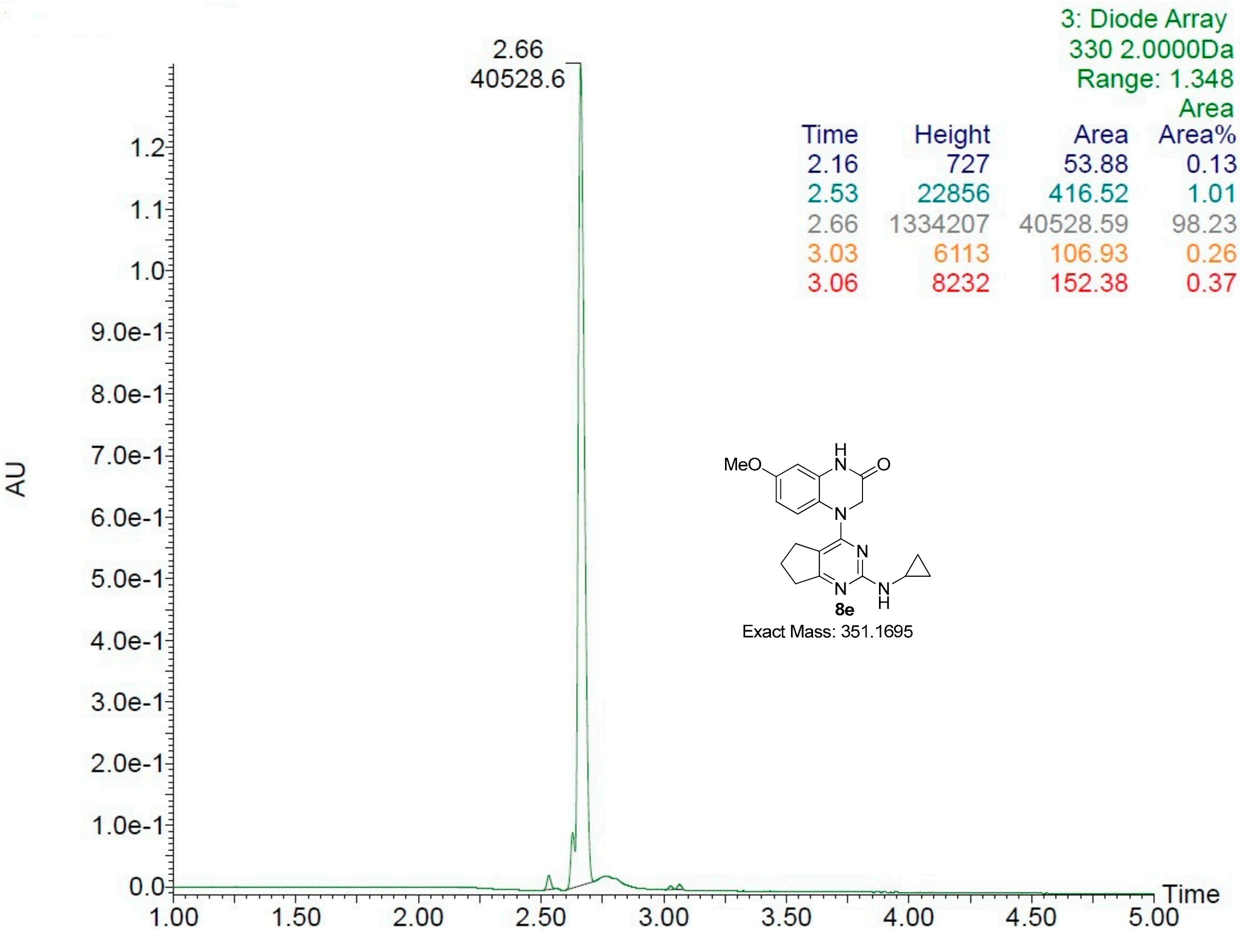

Time	Height	Area	Area%
2.16	727	53.88	0.13
2.53	22856	416.52	1.01
2.66	1334207	40528.59	98.23
3.03	6113	106.93	0.26
3.06	8232	152.38	0.37

HRMS of compound 8e:

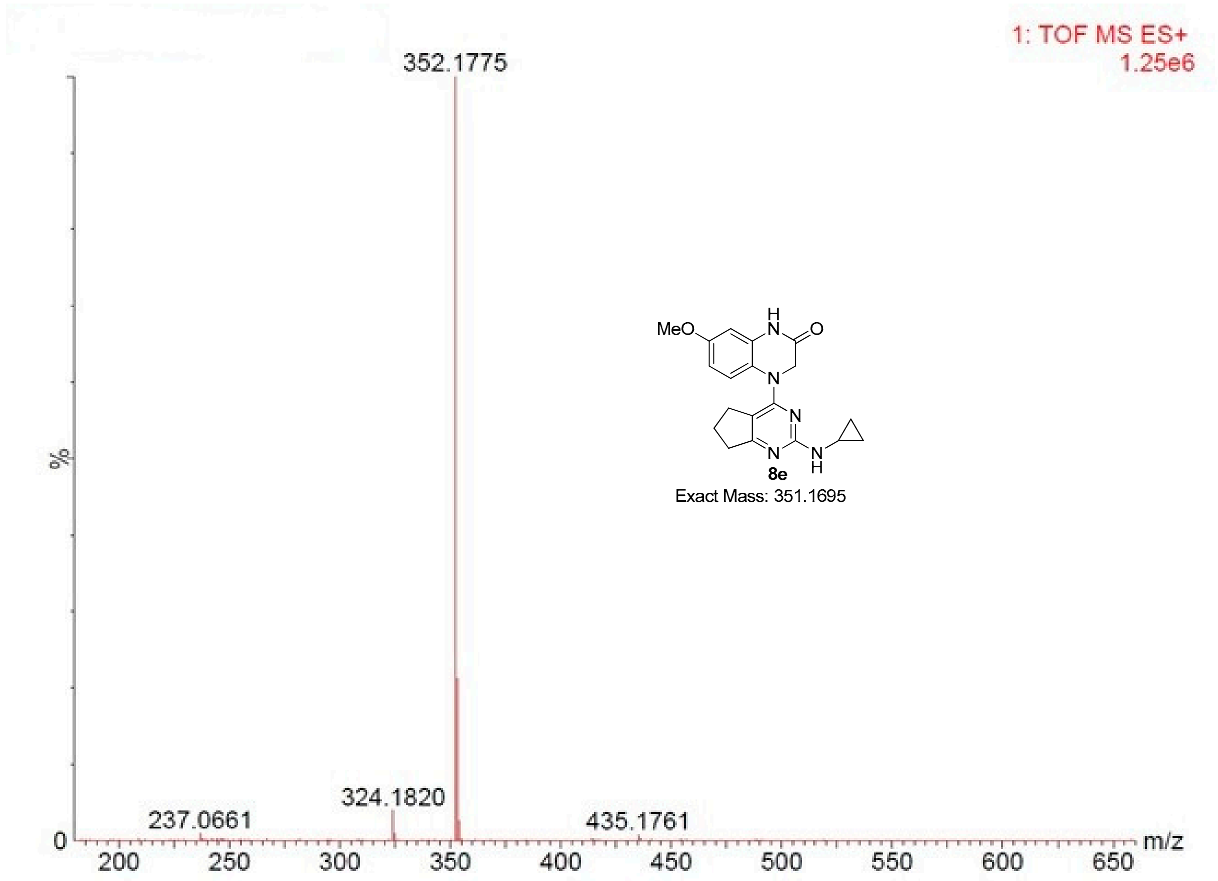

^{1}H-NMR of compound 8f:

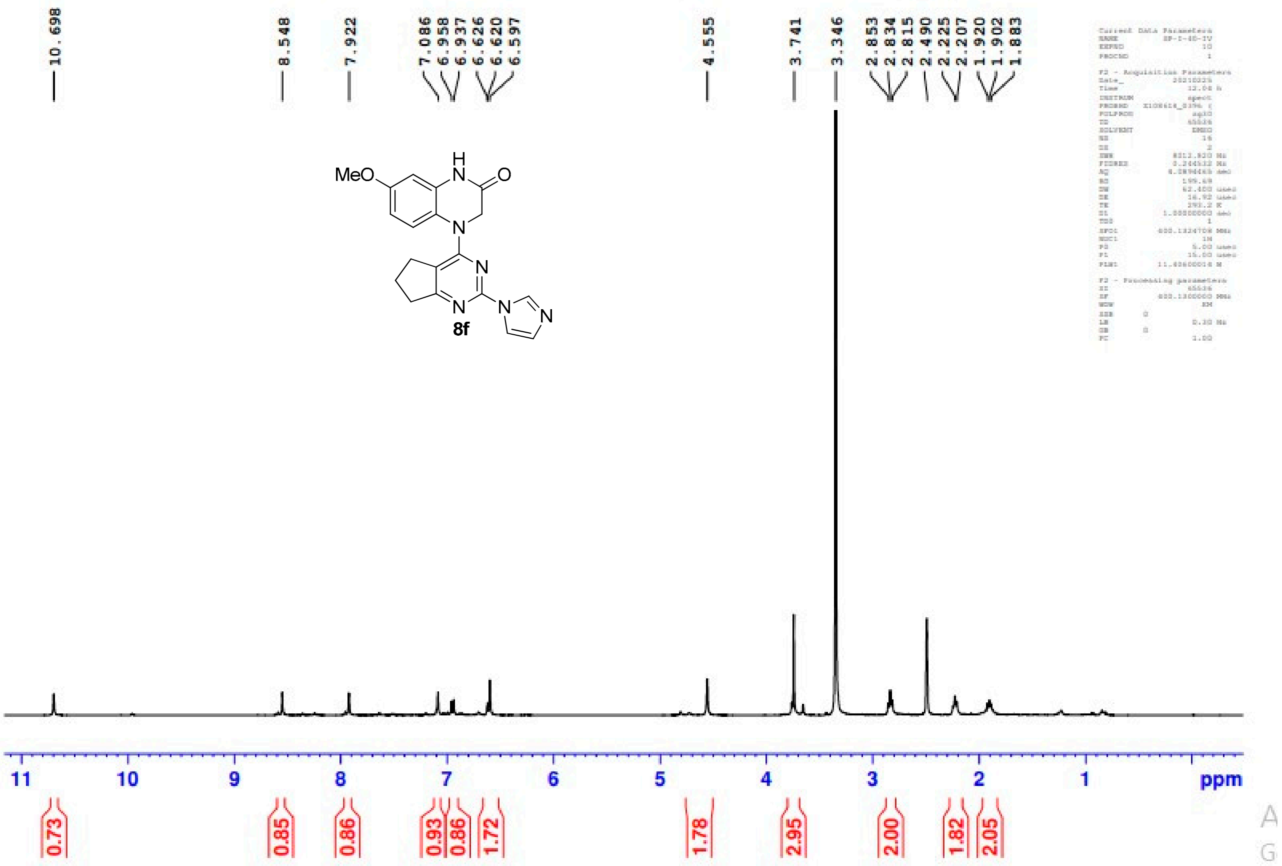

^{13}C-NMR of compound 8f:

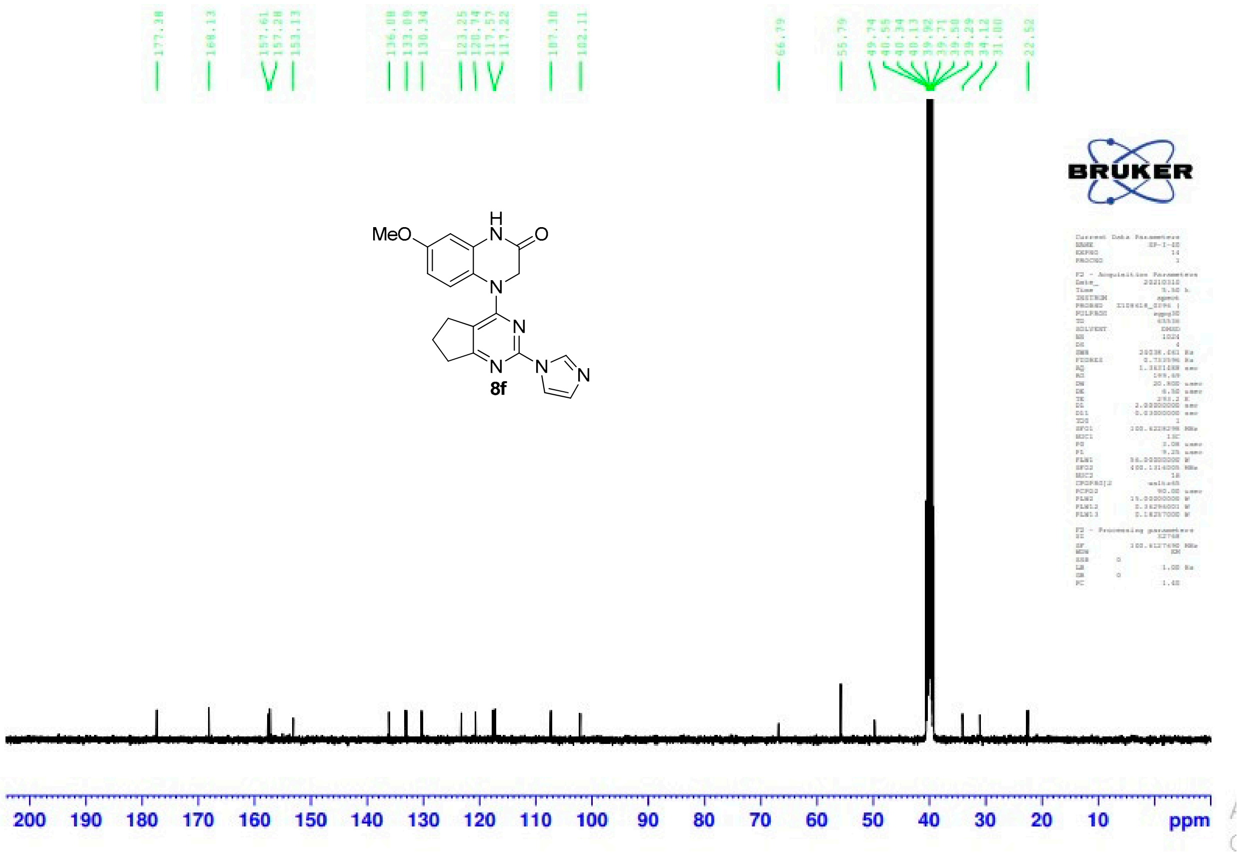

HPLC of compound 8f:

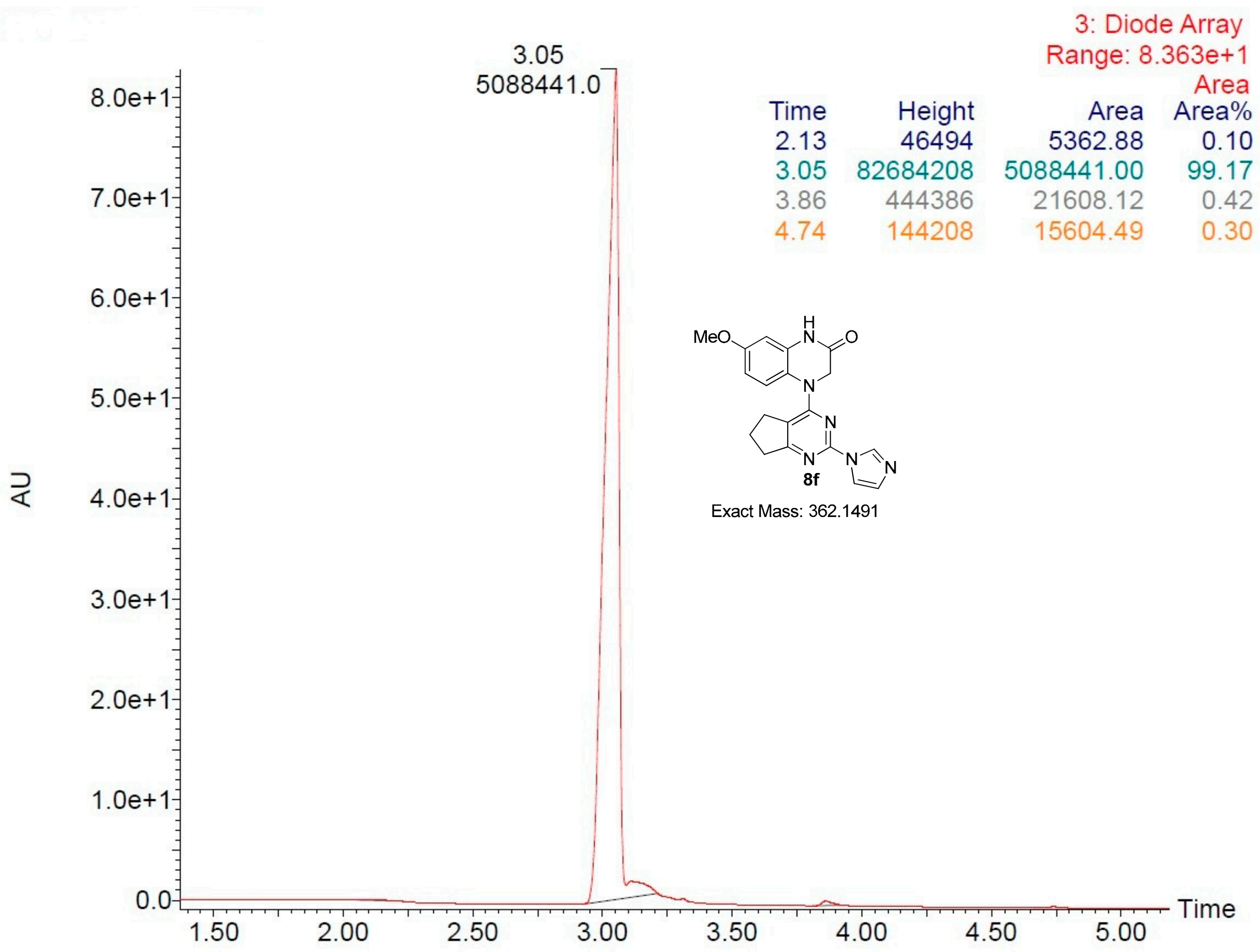

HRMS of compound 8f:

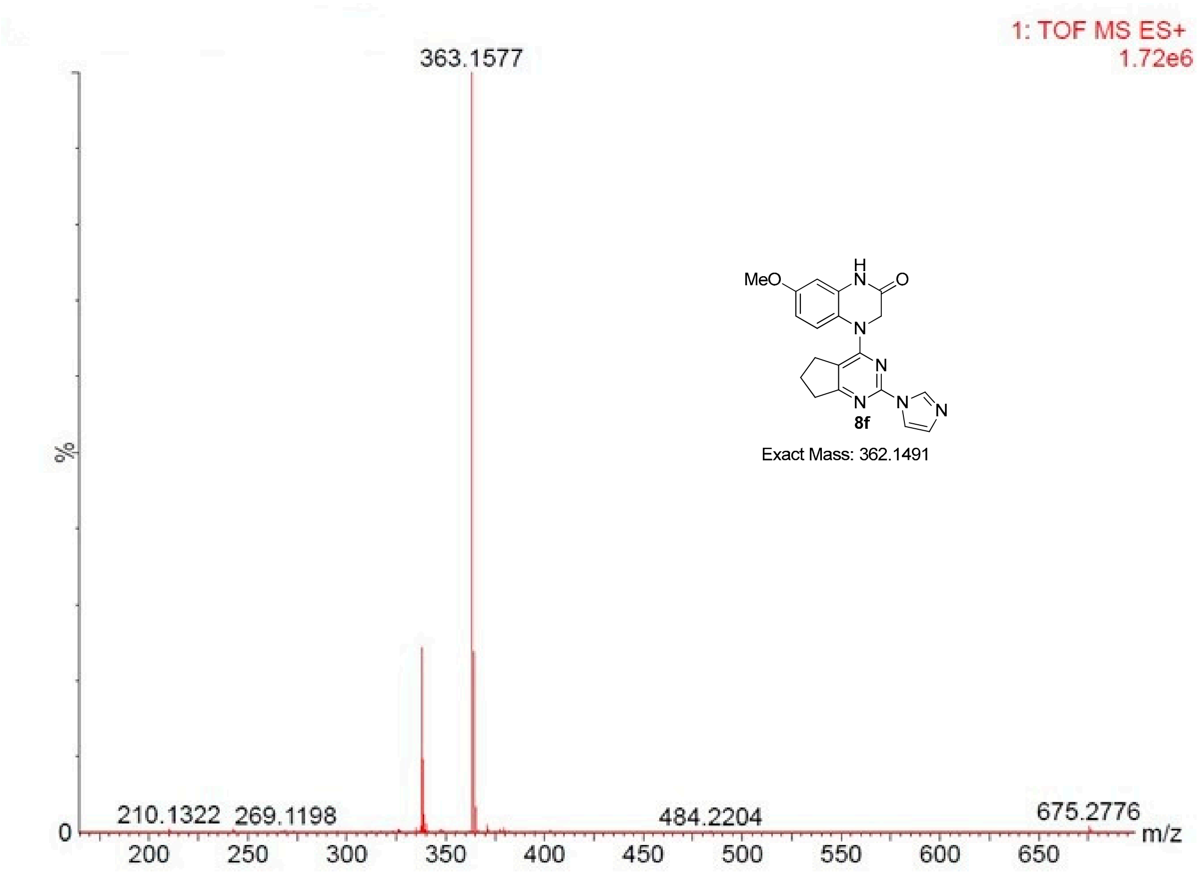

^{1}H-NMR of compound 8g:

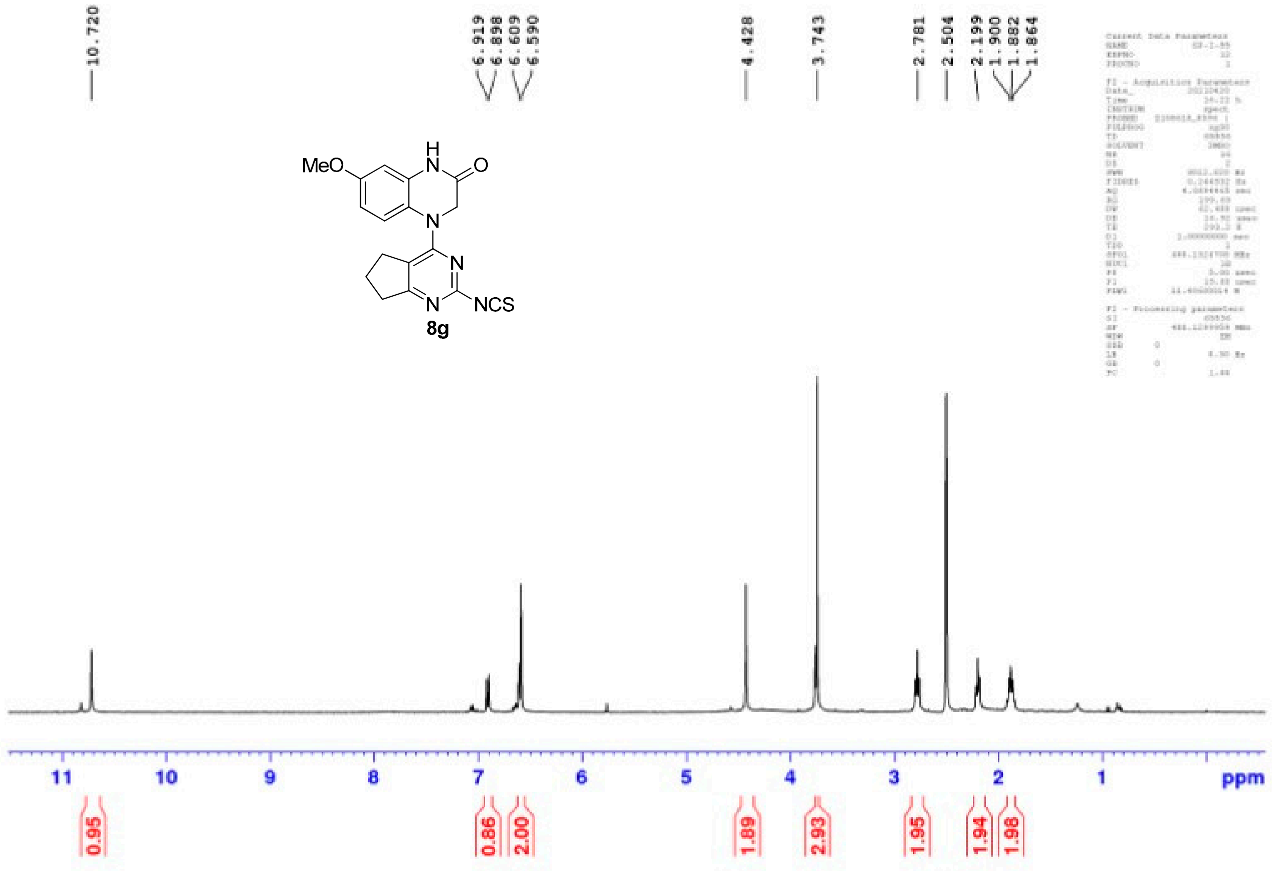

^{13}C-NMR of compound 8g:

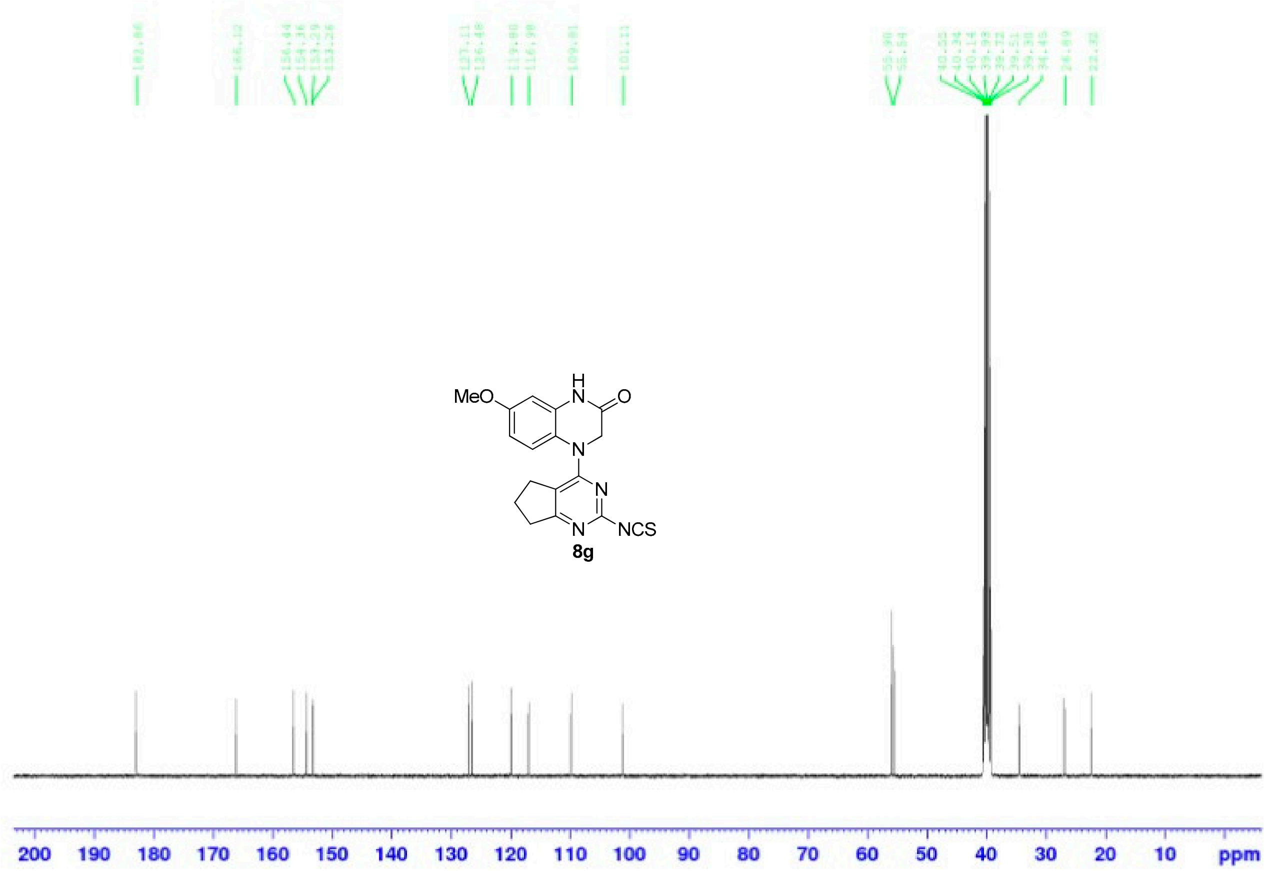

HPLC of compound 8g:

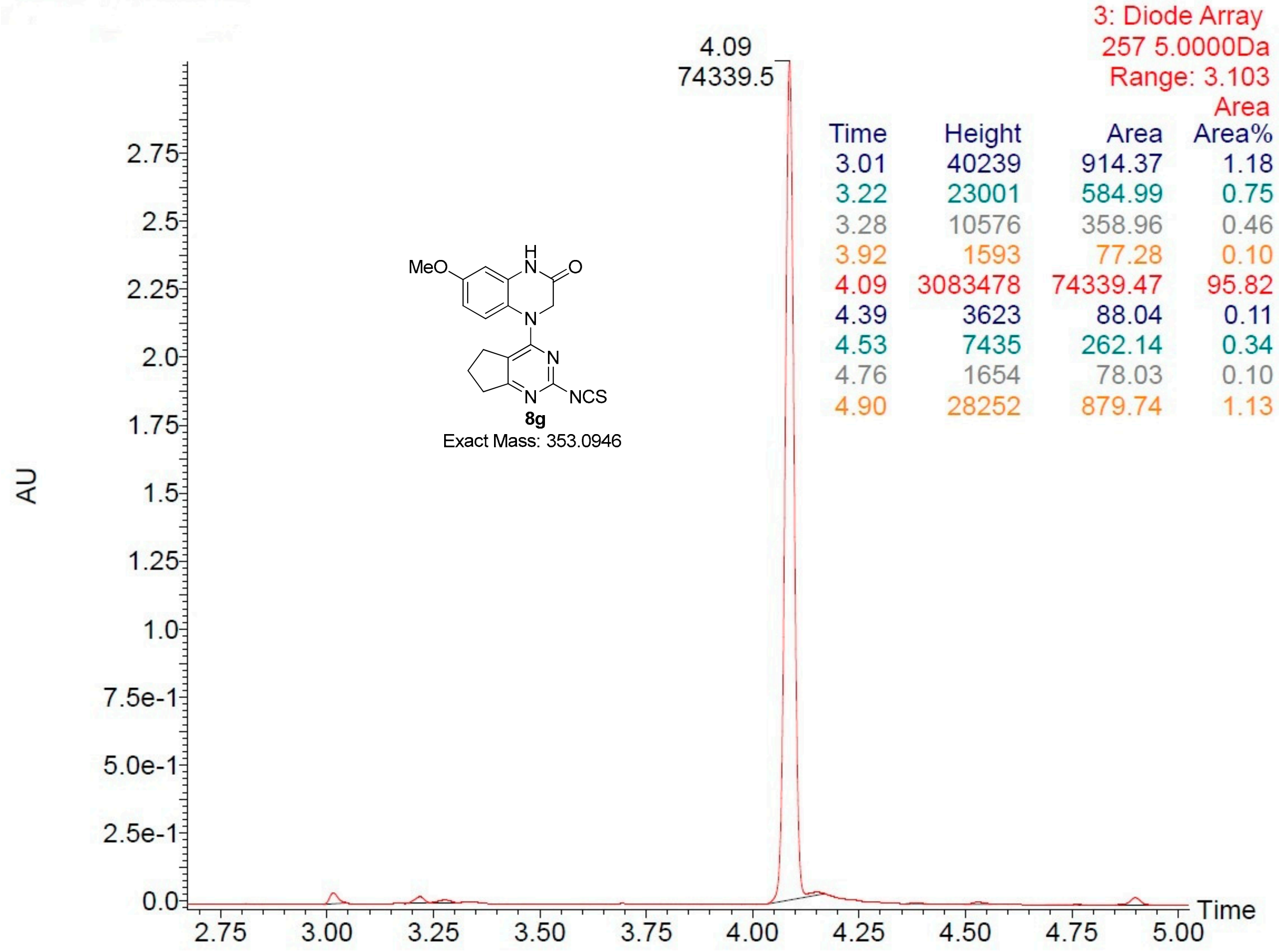

HPLC of compound 8g:

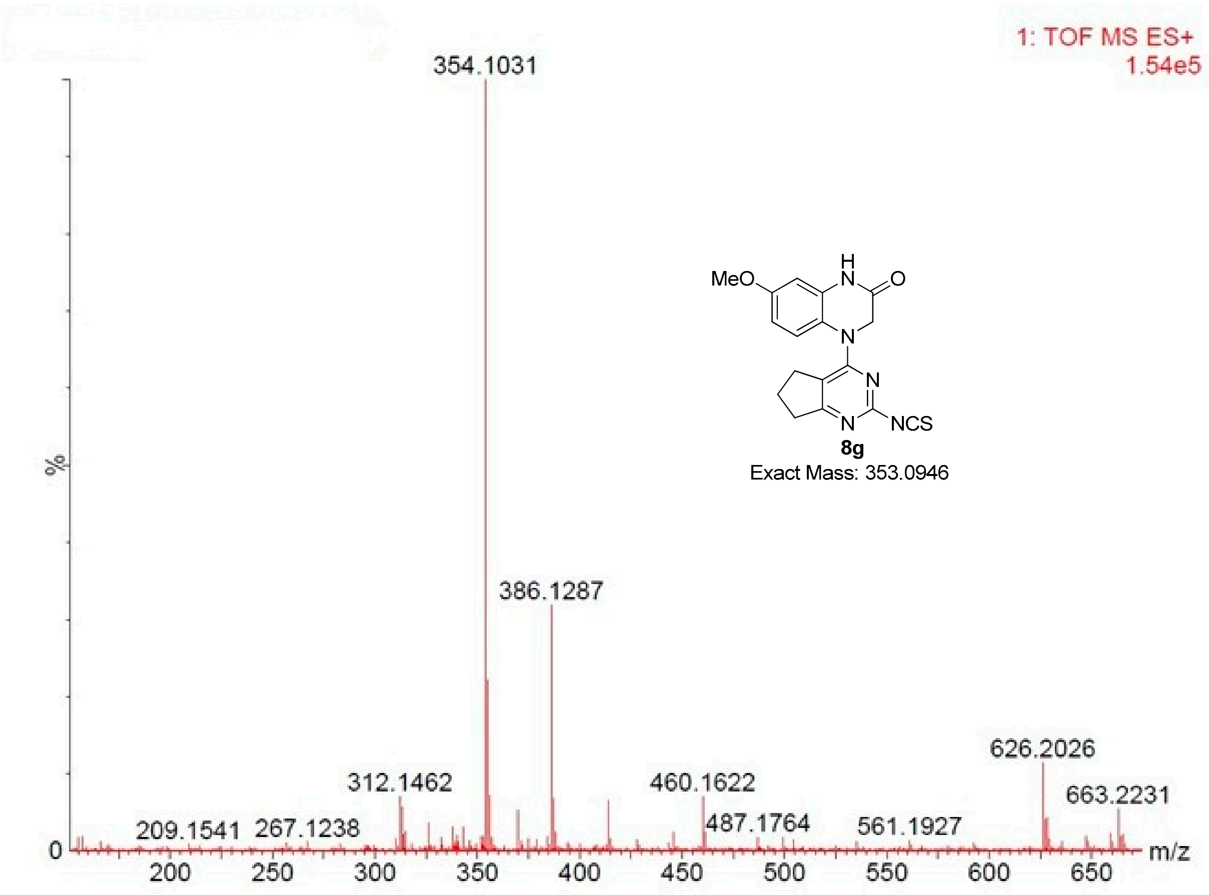

^{1}H-NMR of compound 12:

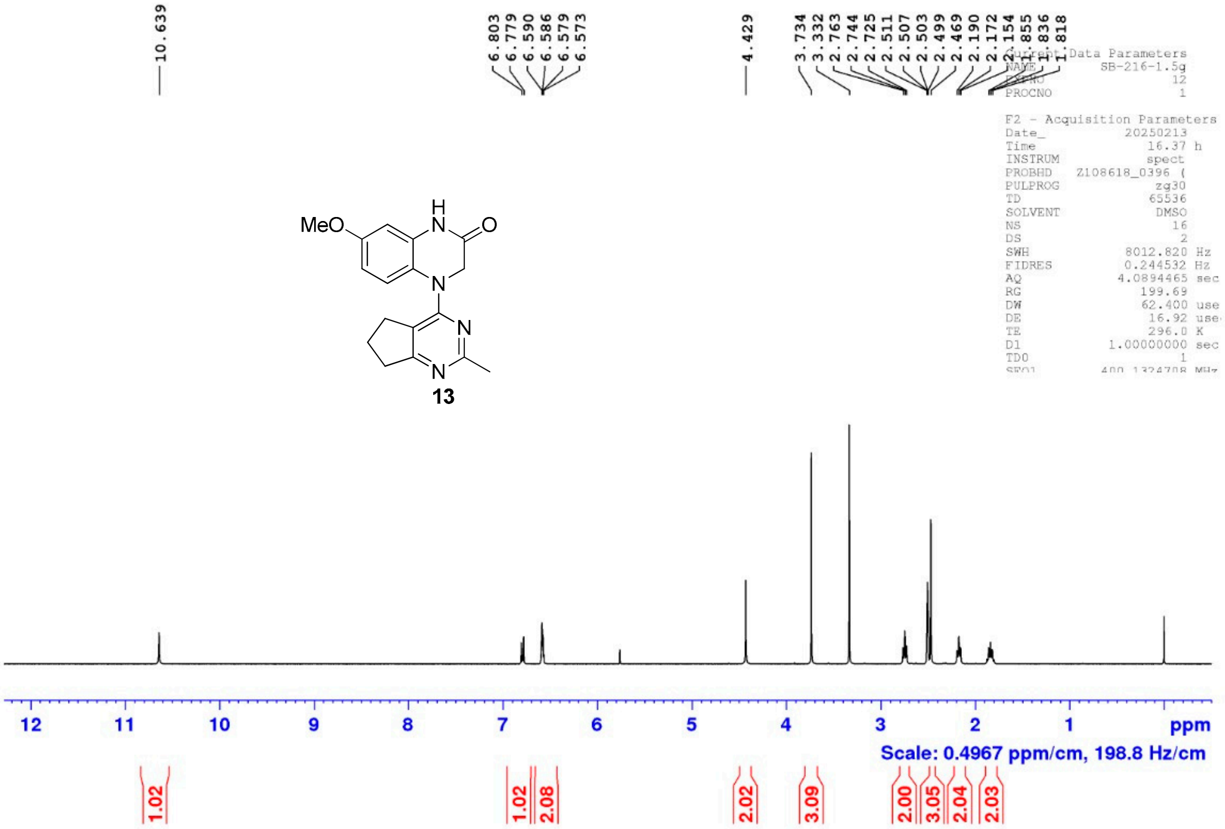

^{13}C-NMR of compound 12:

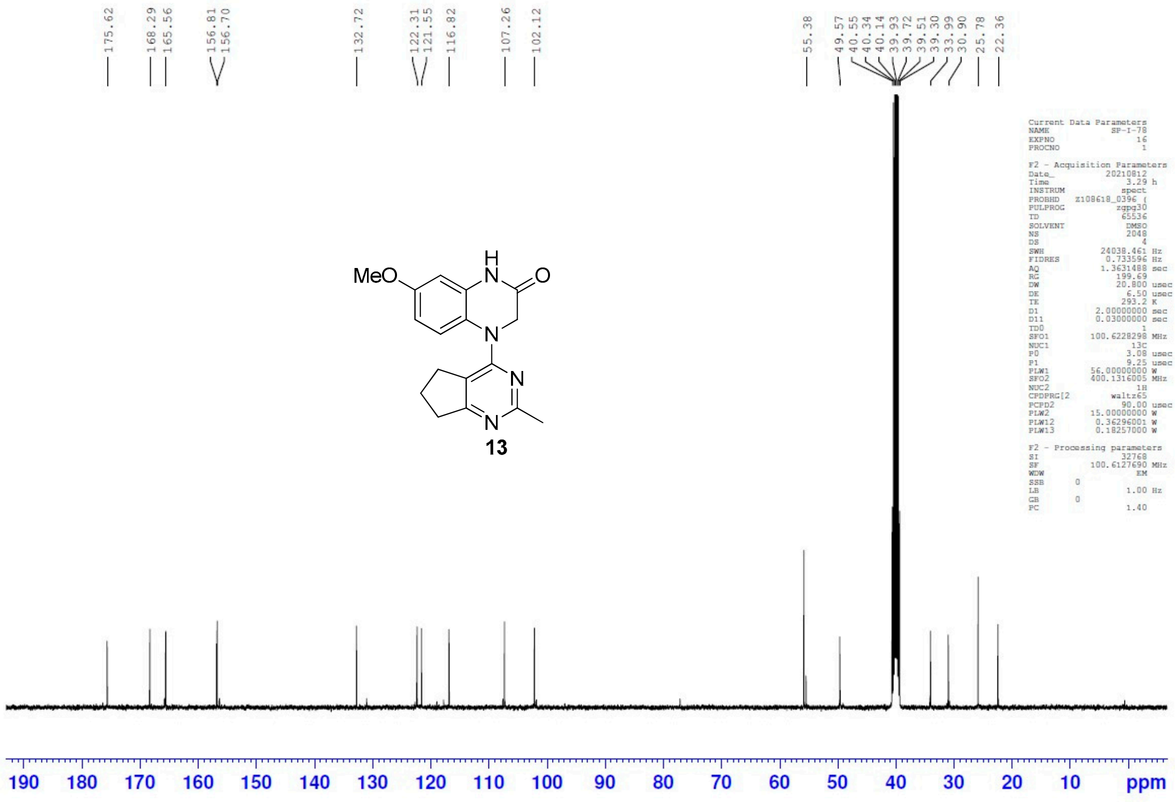

HPLC of compound 12:

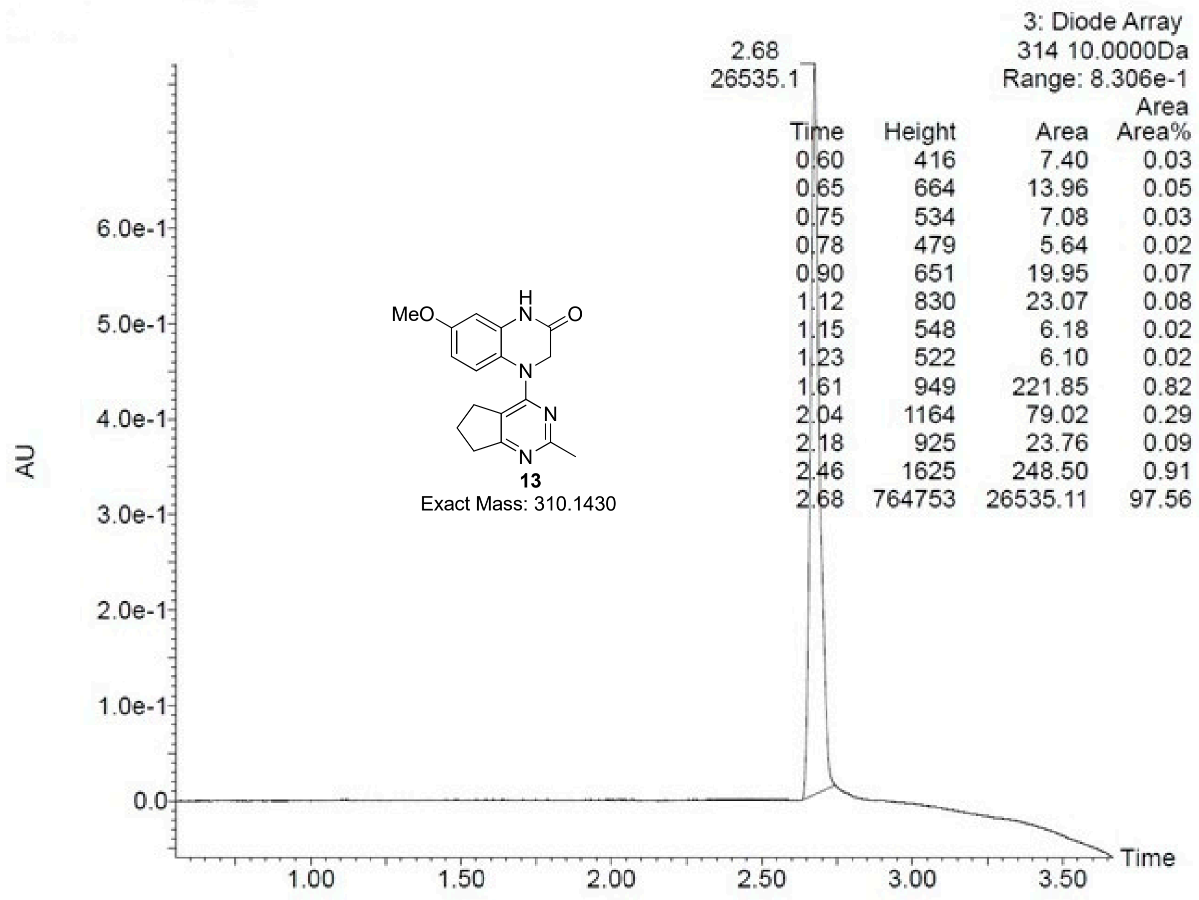

HRMS of compound 12:

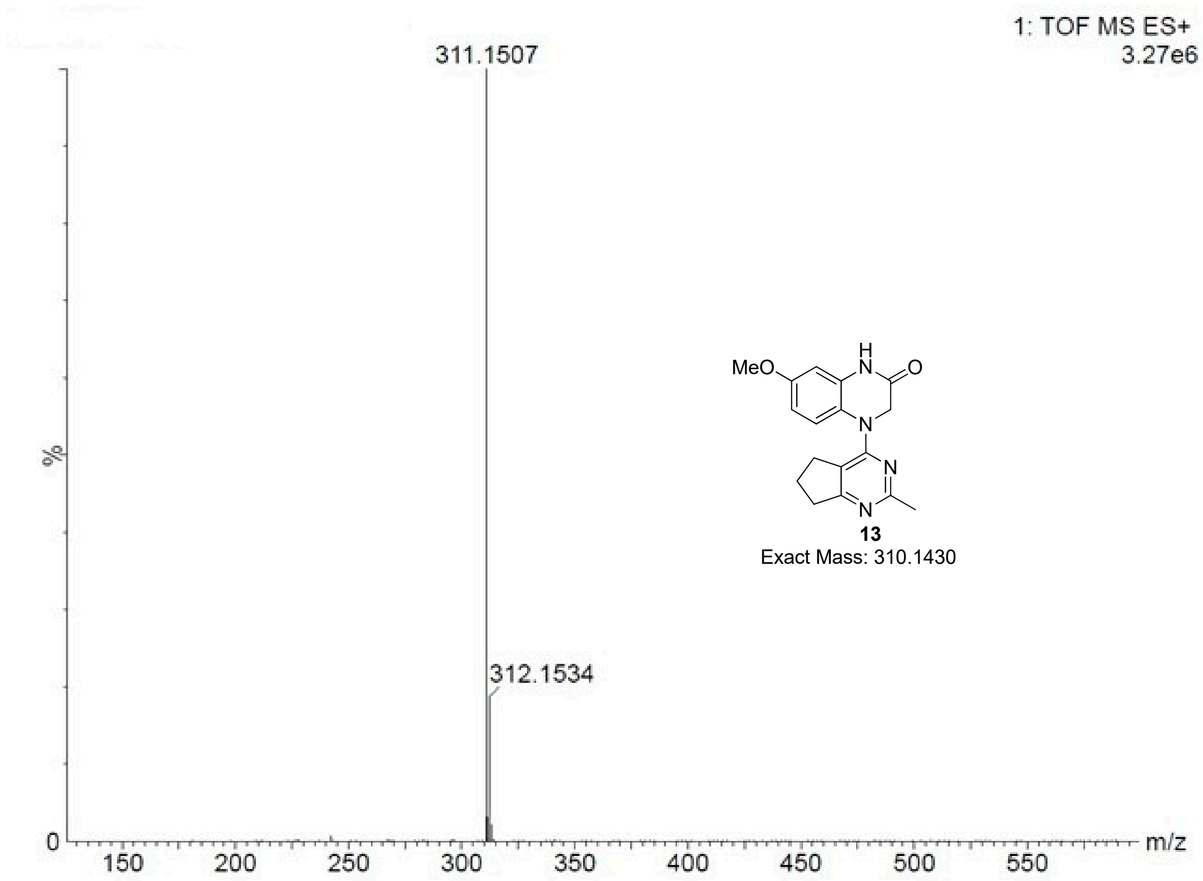

1: TOF MS ES+
3.27e6
311.1507
312.1534
%
0
150
200
250
300
350
400
450
500
550
m/z
MeO
H
N
O
N
N
N
13
Exact Mass: 310.1430
